# DNA methylation-based profiling reveals distinct clusters with survival heterogeneity in high-grade serous ovarian cancer

**DOI:** 10.1186/s13148-021-01178-3

**Published:** 2021-10-13

**Authors:** Jieyu Wang, Jun Li, Ruifang Chen, Huiran Yue, Wenzhi Li, Beibei Wu, Yang Bai, Guohua Zhu, Xin Lu

**Affiliations:** 1 Department of Gynecology, Obstetrics and Gynecology Hospital, Fudan University, No. 128, Shenyang Road, Yangpu District, Shanghai, 200090 China; 2 Shanghai Key Laboratory of Female Reproductive Endocrine-Related Disease, Fudan University, Shanghai, China

**Keywords:** Ovarian cancer, High-grade serous ovarian cancer, DNA profiling, Methylation subtypes, Prognosis

## Abstract

High-grade serous ovarian cancer (HGSOC) is the most common type of epigenetically heterogeneous ovarian cancer. Methylation typing has previously been used in many tumour types but not in HGSOC. Methylation typing in HGSOC may promote the development of personalized care. The present study used DNA methylation data from The Cancer Genome Atlas database and identified four unique methylation subtypes of HGSOC. With the poorest prognosis and high frequency of residual tumours, cluster 4 featured hypermethylation of a panel of genes, which indicates that demethylation agents may be tested in this group and that neoadjuvant chemotherapy may be used to reduce the possibility of residual lesions. Cluster 1 and cluster 2 were significantly associated with metastasis genes and metabolic disorders, respectively. Two feature CpG sites, cg24673765 and cg25574024, were obtained through Cox proportional hazards model analysis of the CpG sites. Based on the methylation level of the two CpG sites, the samples were classified into high- and low-risk groups to identify the prognostic information. Similar results were obtained in the validation set. Taken together, these results explain the epigenetic heterogeneity of HGSOC and provide guidance to clinicians for the prognosis of HGSOC based on DNA methylation sites.

**Supplementary Information:**

The online version contains supplementary material available at 10.1186/s13148-021-01178-3.

## Background

Epithelial ovarian cancer (EOC) has the highest fatality rate of female reproductive cancers [1], primarily because more than 70% of EOCs are diagnosed at advanced stages and are associated with disseminated intraperitoneal disease [2]. EOC is a heterogeneous disease that includes several subtypes with different clinical and molecular features [3]. The most prevalent histotype of EOC is high-grade serous ovarian cancer (HGSOC), which is associated with a poor prognosis.

Epigenetic alterations have recently emerged as a common hallmark of human cancer [4–6]. DNA methylation is a core type of epigenetic alterations and a key epigenetic regulator of gene expression associated with different tumour types [7]. DNA methylation also acts as a biomarker for the prognosis of cancer [8, 9]. Novel methods for tumour classification have been examined in recent years based on genome-wide DNA methylation data [10, 11]. Several DNA methylation pattern analyses have been reported for many cancer types using The Cancer Genome Atlas (TCGA) data [12–16]. There is also increasing interest in the role of DNA methylation in defining molecular subtypes to assist in elucidating the clinical characteristics and prognosis of EOC [17–19]. However, previous researches on the methylation of HGSOC are limited to appointed methylation sites or are established based on the relationship between multiple data integrations. A comprehensive and independent profile of the prognostic value of these aberrantly methylated biomarkers in the HGSOC subtype is not clear. Therefore, a comprehensive and independent assessment of DNA methylation is needed to better understand the methylation-based classification of HGSOC subtypes.

In the present population-based study, we aimed to investigate whether clinically relevant and different HGSOC subtypes could be distinguished using genome-wide DNA methylation pattern evaluation. We examined HGSOC classification strategies based on DNA methylation profiles from the TCGA database. Our methylation-based classification system improves the understanding of methylation heterogeneity and may lead to the identification of prognostic signatures and provide good practice guidelines for the clinical treatment of HGSOC patients.

## Methods

### Methylation data downloading and processing

**Fig. 1:**
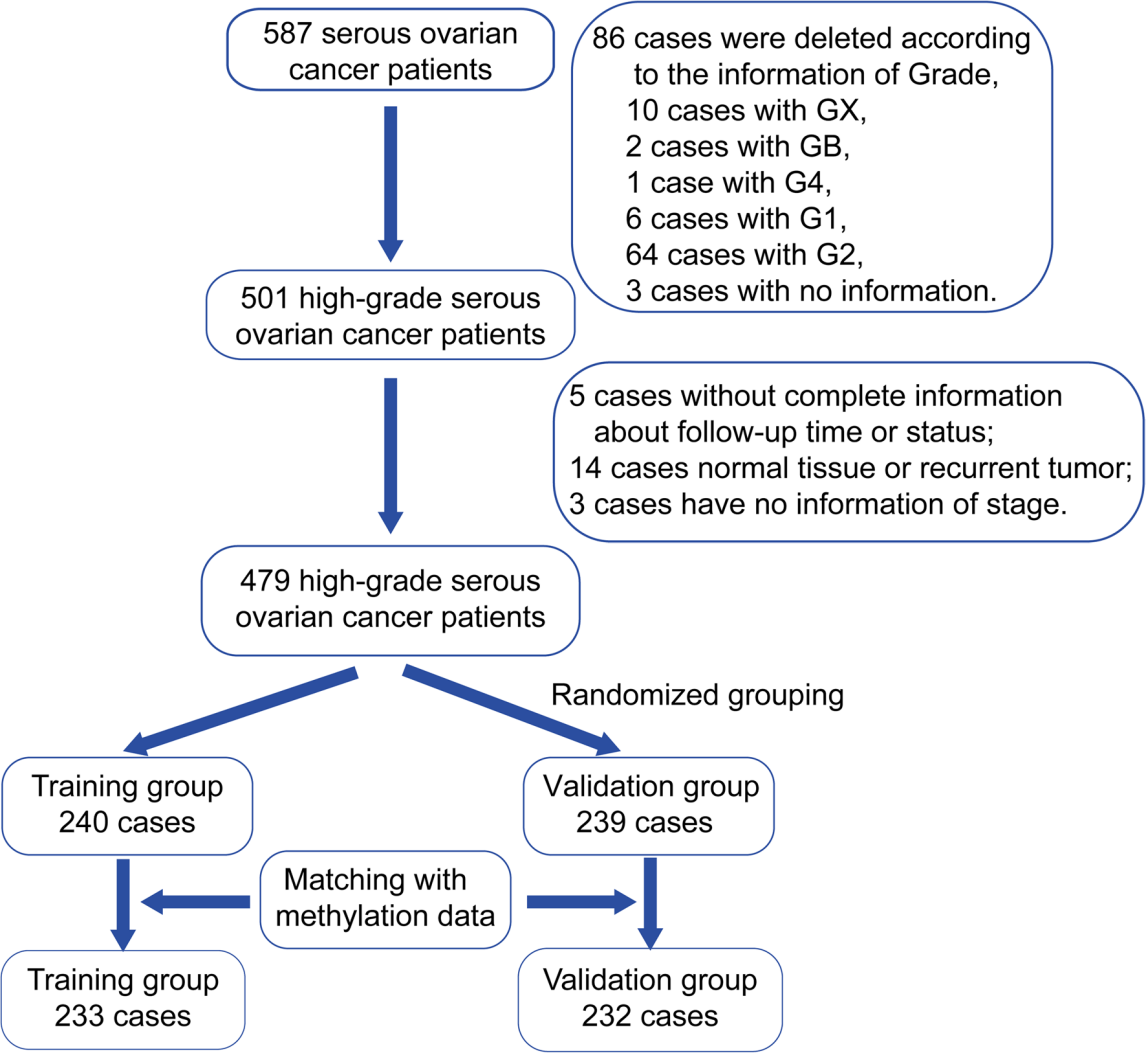
Flow chart describing the cases analysed in the study

HGSOC methylation data and clinical follow-up data of HGSOC patients were downloaded from TCGA. These data were collected and analysed in accordance with the TCGA Human Subjects Protection and Data Access Policies. The methylation level of each probe was represented by the β-value, which ranges from 0 (unmethylated) to 1 (fully methylated). To obtain more precise classification results, we deleted some methylation loci using the following exclusion criteria: (1) cytosines preceding guanosine sites (CpGs) in sex chromosomes and (2) missing data in more than 70% of the samples. The remaining missing probes were imputed using the *k*-nearest neighbours [20]. DNA methylation in promoter regions (2 kb upstream to 0.5 kb downstream from transcription start sites) strongly influences gene expression. Therefore, we annotated these sites into promoter regions and reached a final matrix with 21,123 probes. By incorporating patient clinical information and integrating the DNA methylation array data, the ComBat algorithm in the sva R package was used to remove batch effects. From TCGA, we obtained clinical follow-up information for 587 serous ovarian cancer patients and excluded samples that did not meet the following inclusion criteria: (1) pathology limited to serous ovarian cancer, (2) disease was grade 3, (3) available survival time data, (4) primary tumour sample type, (5) available stage information, and (6) available age information. A total of 479 patients were ultimately included. The samples were randomly divided into a training group (*n* = 240) and a validation group (*n* = 239). Survival times less than 30 days were generally due to operative complications, and these samples were deleted. Samples without methylation information were also excluded from our study. Finally, there were 233 samples in the training group and 232 samples in the validation group (Fig. 1).

### Determination of statistically significant methylation sites with Cox proportional risk regression models

Methylation sites associated with prognosis were regarded as classification characteristics. The methylation profiles and corresponding clinical characteristic data of the training set were used to construct the Cox regression model with the R package “survival”, and the methylation level of each site was analysed according to the following steps: (a) a univariate Cox proportional hazard regression model was constructed with *p* < 0.05; (b) the significant methylation sites obtained from univariate Cox regression models were used to establish the multivariate Cox proportional risk regression model based on age and stage as the covariants in survival analysis; (c) statistically significant sites (*p* < 0.05) in multivariate analyses were used as classification features, and the coxph function in survival package R was used.

### Gene ontology (GO) and Kyoto Encyclopedia of Genes and Genomes (KEGG) analysis annotated by the CpG sites

The methylation sites that were statistically significant in multivariate analyses were annotated to the corresponding genes. The “DOSE”, “clusterProfiler” and “enrich plot” R packages were used to complete the GO and KEGG analyses. A maximum *p* value of 0.05 was chosen to select only significantly enriched categories.

### Consensus clustering analysis based on methylation profiles to obtain methylation subtypes associated with HGSOC prognosis

Consensus Clustering Plus [21, 22] is an appropriate method to examine new subclasses of cancer. The present study performed clustering based on the previously obtained methylation sites that were significant in multivariate analyses with the Consensus Cluster Plus algorithm [22] of R software to determine the methylation subgroups of HGSOC. The similarity distance between samples was calculated by the Euclidean distance method [23], and *κ*-means [24] was used as the clustering algorithm to search for reliable and stable subgroup classifications. After executing Consensus Cluster Plus, consensus clustering results were obtained. The optimal cluster number was determined based on the following criteria: a relatively low-variation coefficient within the cluster, relatively high consistency, and no obviously increased area under the cumulative distribution function curve. The coefficient of variation was calculated with the following formula: CV = (SD/MN) * 100%, in which MN represents the mean of samples and SD represents the standard deviation.

### Survival and clinical characteristic analyses

The log-rank test and Kaplan–Meier plots were used to determine the overall survival and significant differences between HGSOC methylation clusters. Associations between clinical characteristics and HGSOC methylation clusters were analysed with the chi-square test. All tests were two-sided, and differences with *p* < 0.05 were considered significant.

### Construction of the prognostic model and verification using fivefold cross-validation and the validation data

The prognosis-related CpG sites were selected to establish the Cox proportional hazards model. Cox regression analysis was performed using multivariate Cox hazards regression model with stepwise method, which was implemented using survival package in R project. A risk score formula for predicting OS was developed based on a linear combination of the expression level multiplied regression coefficient derived from the cox regression model:

$${\text{Risk}}\;{\text{score}} = {\text{Exp}}_{{{\text{methy}}\_{\text{loci}}1}} \times \beta_{{{\text{methy}}\_{\text{loci}}1}} + {\text{ Exp}}_{{{\text{methy}}\_{\text{loci}}2}} \times \beta_{{{\text{methy}}\_{\text{loci}}2}} + \cdots {\text{Exp}}_{{{\text{methy}}\_{\text{locin}}}} \times \beta_{{{\text{methy}}\_{\text{locin}}}}$$

(where “Exp” denotes the methylation level of loci and “β” represents the regression coefficient from the multivariate Cox regression model [25, 26]). By utilizing the median risk score as the threshold, the HGSOC patients were stratified into high- and low-risk groups. Survival curves were estimated by the Kaplan–Meier and log-rank method. Fivefold cross-validation (CV) was used on the patients. The same processes were performed on the validation data to verify the stability and applicability of the model. R/Bioconductor tools and R functions were used for all analyses in R version 3.6.1.

## Results

### Clinicopathological features and the initial screening of DNA methylation loci of HGSOC

Table 1 summarizes the clinicopathological characteristics of the training group and validation group. The mean age of all patients was 59 years. A total of 436 patients (93.8%) had advanced disease, and 58.1% achieved complete remission. 66.4% of the patients underwent optimal cytoreductive surgery. However, the integrity of information for venous invasion and lymphatic invasion was not satisfactory. Clinicopathological parameters were well balanced between the training and the validation groups (Table 1, *p* > 0.05). A univariate Cox proportional hazard regression model was used to distinguish the 1434 methylation sites that significantly correlated with survival (*p* < 0.05). Age and stage are universally acknowledged factors associated with HGSOC prognosis. Therefore, age, stage, and 1434 methylation sites were added to the multivariate Cox proportional hazard regression model, which revealed 780 sites that were significantly related to survival (Additional file 2: Table S1).

**Table 1:** The demographic and clinicopathological parameters between training group and validation group

	*N*	Training group	Test group	*p* Value
Total, *n*	465	233(50.1%)	232(49.9%)	
Mean age, y		59.61 ± 11.03	59.84 ± 11.54	0.822
*Stage*				0.939
I	12(2.6%)	7(3.0%)	5(2.2%)	
II	17(3.7%)	8(3.4%)	9(3.9%)	
III	369(79.4%)	185(79.4%)	184(79.3%)	
IV	67(14.4%)	33(14.2%)	34(14.7%)	
*Primary therapy outcome*				0.773
Complete remission	270(58.1%)	140(60.1%)	130(56.0%)	
Partial remission	51(11.0%)	25(10.7%)	26(11.2%)	
Progressive disease	32(6.9%)	14(6.0%)	18(7.8%)	
Stable disease	27(5.8%)	15(6.4%)	12(5.2%)	
Unknown	85(18.3%)	39(16.7%)	46(19.8%)	
*Tumour residual*				0.894
No macroscopic disease	100(21.5%)	50(21.5%)	50(21.6%)	
1–10 mm	209(44.9%)	100(42.9%)	109(47.0%)	
11–20 mm	28(6.0%)	15(6.4%)	13(5.6%)	
> 20 mm	89(19.1%)	48(20.6%)	41(17.7%)	
Unknown	39(8.4%)	20(8.6%)	19(8.2%)	
*Venous invasion*				0.296
No	53(11.4%)	29(12.4%)	24(10.3%)	
Yes	72(15.5%)	41(17.6%)	31(13.4%)	
Unknown	340(73.1%)	163(70.0%)	177(76.3%)	
*Survival status*				0.365
Alive	188(40.4%)	99(42.5%)	89(38.4%)	
Dead	277(59.6%)	134(57.5%)	143(61.6%)	
*Lymphatic invasion*				0.795
No	62(13.3%)	33(14.2%)	29(12.5%)	
Yes	117(25.2%)	60(25.8%)	57(24.6%)	
Unknown	286(61.5%)	140(60.1%)	146(62.9%)	

### Enrichment analysis of differentially methylated genes

**Fig. 2:**
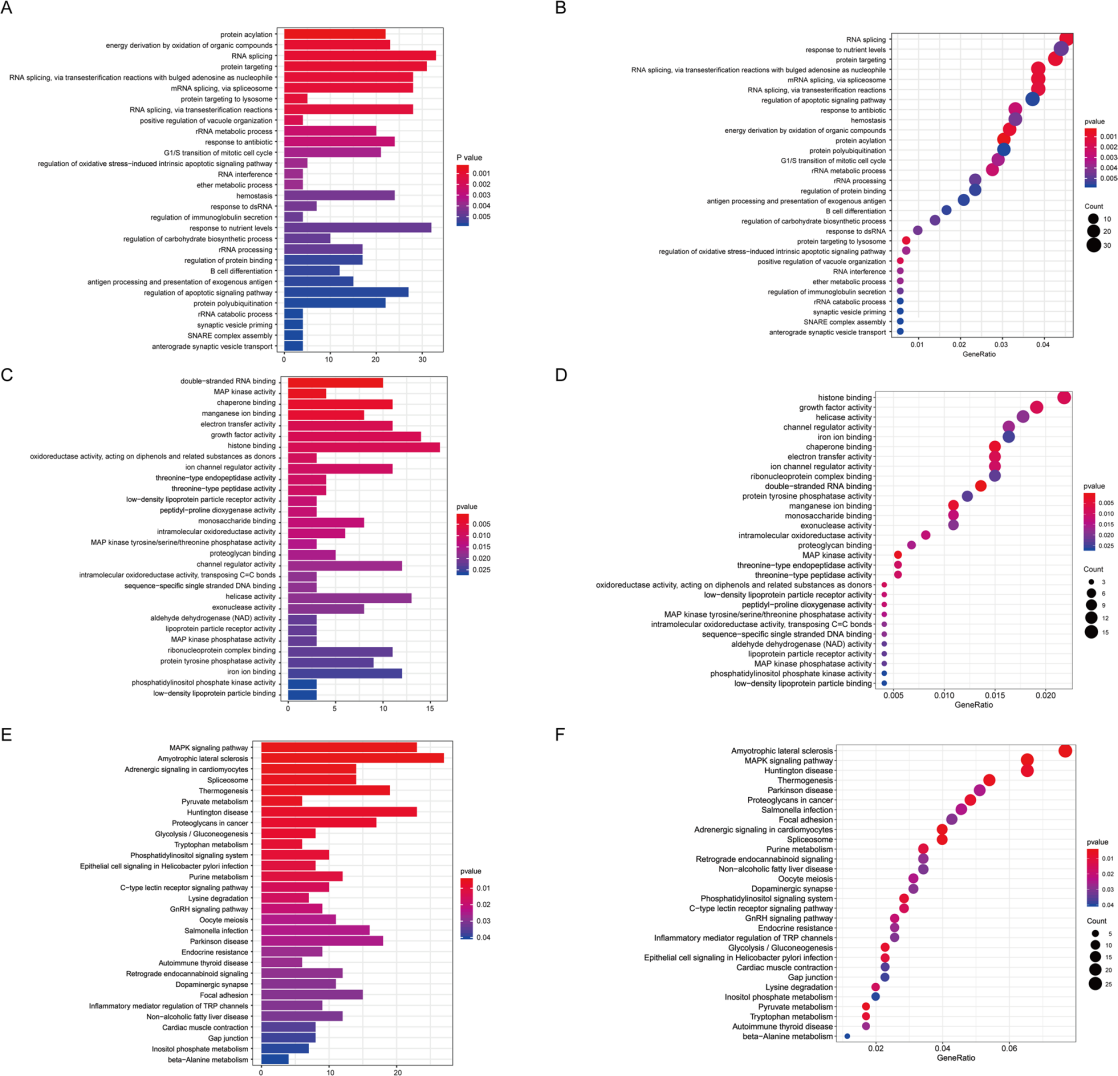
Gene ontology (GO) and KEGG (Kyoto Encyclopedia of Genes and Genomes) pathway analyses were based on corresponding genes derived from the differentially methylated probe (Additional file 2: Table S1). The GO terms included biological processes (**A** and **B**) and molecular functions (**C** and **D**). **E** and **F** show the related pathway analysed by KEGG. The results are shown as bar plots (**A**, **C** and **E**) and bubble plots (**B**, **D** and **F**) generated with the R package

It is important to understand the molecular mechanisms of genes associated with methylation loci. To examine possible interactions between differentially methylated genes and their possible influence, we analysed the 780 sites that were significantly correlated with survival in the multivariate Cox proportional hazard regression model and examined their co-expressed genes. We performed GO (Fig. 2A–D) and KEGG pathway analyses (Fig. 2E, F). Biological significance (Fig. 2A, B, Additional file 3: Table S2) revealed enrichment in protein acylation, energy derivation by oxidation, and G1/S transition of the mitotic cell cycle. We also performed molecular function analysis and found that mitogen-activated protein kinase (MAPK) activity, growth factor activity, histone binding, and oxidoreductase activity were involved in the network (Fig. 2C, D, Additional file 4: Table S3). Pathways related to MAPK signalling, pyruvate metabolism, and glycolysis were enriched in the KEGG analysis (Fig. 2E, F, Additional file 5: Table S4). The methylation loci and corresponding genes involved in different pathways are shown in Additional file 6: Table S5. The results suggest that the methylation sites examined in this study may affect HGSOC tumorigenesis and development via MAPK signalling, pyruvate metabolism, and glycolysis.

### Consensus clustering revealed distinct methylation subtypes associated with HGSOC prognosis

**Fig. 3:**
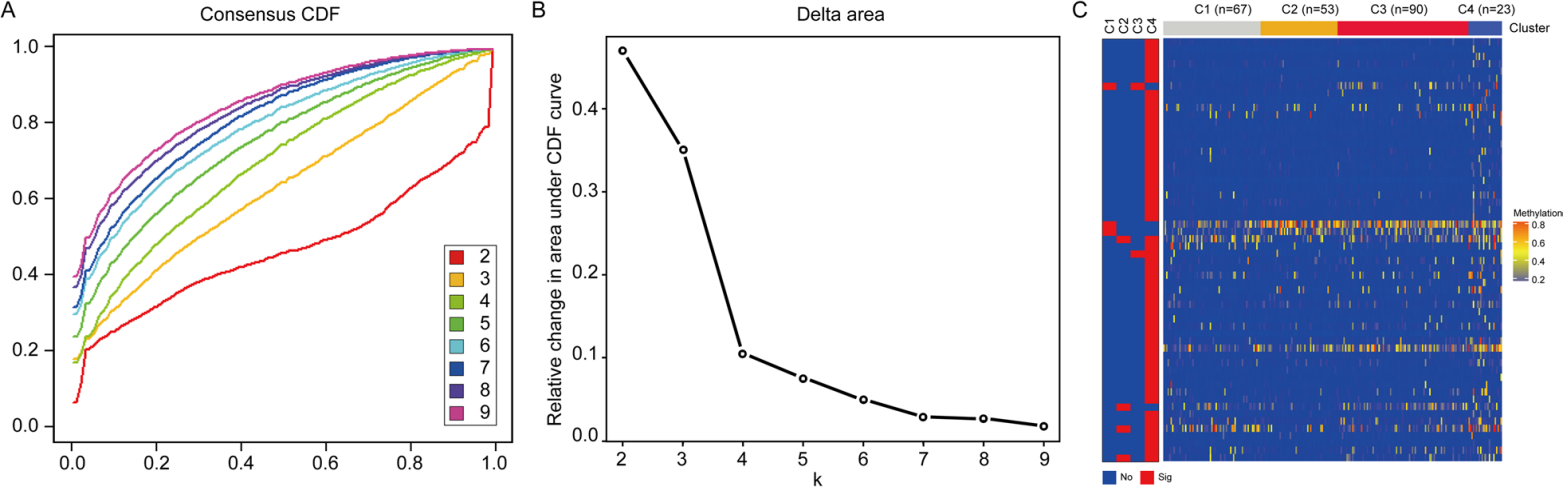
Consensus clustering for DNA methylation of HGSOC. **A** Consensus cumulative distribution function (CDF) plot. The CDF plot shows the cumulative distribution functions of the consensus matrix for each k (indicated by colours). **B** Delta area plot. This graph shows the relative change in area under the CDF curve. In *k* = 4, the shape of the curve approaches the ideal step function, and the shape hardly changes as we increase *K* past 4. Therefore, four clusters were chosen as the optimal number. **C** Heat map generated using the pheatmap function with DNA methylation classification. The left bar represents the significantly different DNA methylation loci (Table 2)

The Consensus Cluster Plus R software package was used to analyse the consensus clustering of 233 samples. The delta area under the curve had an appreciable increase from *k* = 2 to *k* = 4, but the increase rate was not obvious for *k* > 4 (Fig. 3A, B). Therefore, 4 was selected as a suitable cluster number for further analysis in this study. When *k* = 4, we named the resulting subgroups C1 (28.8%; 67 samples), C2 (22.7%; 53 samples), C3 (38.6%; 90 samples), and C4 (9.9%; 23 samples). By identifying DNA methylation panels that discriminated the four subgroups, we found that the methylation levels of the four subgroups were significantly related to different molecular features (Fig. 3C). C4 was the poorest prognostic group and exhibited hypermethylation of 54 methylation loci, which corresponded to 51 genes (Additional file 7: Table S6). Therefore, the 51 genes composed the hypermethylation panel, in which ANXA7, IGF2, and SLC5A8 harboured two hypermethylation sites in the samples from the C4 subgroup. C1 featured hypomethylation of cg03848675, cg12493906, and cg13055001, which were annotated as FOXF2, MMP26, and PPP1CA, respectively. FOXF2 and MMP26 were primarily related to tumour metastasis and invasion. PPP1CA was associated with the MAPK pathway. C3 exhibited hypermethylation of cg03848675, which was opposite to the pattern observed in C1, and exhibited hypomethylation of cg14290451 (RPL10A). C2 had the best prognosis and featured hypomethylation of cg13791131, cg25574024, cg24673765, and cg27239157, which were annotated as IGF2 (cg13791131, cg25574024), HSPB6 (cg24673765), and MCF2L2 (cg27239157). IGF2 plays a key role in glucose metabolism, HSPB6 is associated with insulin resistance [27], and MCF2L2 is related to type 1 diabetes [28] and polycystic ovary syndrome [29]. All of these genes play a role in metabolic disorders. Therefore, the four subgroups classified based on the methylation levels may reflect changes in some molecular genetic features.

### Survival and clinical characteristic analyses of methylation subtypes

We combined the four molecular subtypes with the clinical parameters and performed Kaplan–Meier analysis, and the log-rank tests showed that survival curves of these four molecular subtypes were significantly different (Table 2). C4 was obviously more malignant than the other clusters (Table 2) and exhibited hypermethylation at 54 methylation loci (Additional file 7: Table S6). However, the median overall survival time was significantly longer in C2 than the other clusters (64 months in C2 vs. 35 months in C1, 48 months in C3, 24 months in C4, *p* = 0.0001, Table 2). We also noted that the residual tumour and lymphatic invasion rates were significantly different between the four subgroups (*p* = 0.027 and 0.031, respectively, Table 2). C4 was highly interrelated with larger residual tumours (≥ 11 mm), and the lymphatic invasion rate was the lowest in C2, which partially explains why C4 had the worst prognosis and C2 had the best prognosis (Table 2). Median age did not differ between the four clusters (*p* = 0.774, Table 2).

**Table 2:** Clinicopathologic features of training group stratified by methylation clusters

	*N*	C1	C2	C3	C4	*p* Value
Total, *n*	233	67(28.8%)	53(22.7%)	90(38.6%)	23(9.9%)	
Mean age, y		60.1 ± 11.21	62.34 ± 11.84	57.03 ± 10.12	61.96 ± 10.45	
*Stage*						0.471
I	7(3.0%)	2(3.0%)	2(3.8%)	3(3.3%)	0	
II	8(3.4%)	1(1.5%)	2(3.8%)	4(4.4%)	1(4.3%)	
III	185(79.4%)	53(79.1%)	47(88.7%)	66(73.3%)	19(82.6%)	
IV	33(14.2%)	11(16.4%)	2(3.8%)	17(18.9%)	3(13.0%)	
*Primary therapy outcome*						0.227
Complete remission	140(60.1%)	36(53.7%)	32(60.4%)	62(68.9%)	10(43.5%)	
Partial remission	25(10.7%)	6(9.0%)	4(7.5%)	10(11.1%)	5(21.7%)	
Progressive disease	14(6.0%)	7(10.4%)	2(3.8%)	3(3.3%)	2(8.7%)	
Stable disease	15(6.4%)	4(6.0%)	5(9.4%)	3(3.3%)	3(13.0%)	
Unknown	39(16.7%)	14(20.9%)	10(18.9%)	12(13.3%)	3(13.0%)	
*Tumour residual*						**0.027**
No macroscopic disease	50(21.5%)	12(17.9%)	19(35.8%)	17(18.9%)	2(8.7%)	
1–10 mm	100(42.9%)	36(53.7%)	13(24.5%)	40(44.4%)	11(47.8%)	
11–20 mm	15(6.4%)	3(4.5%)	7(13.2%)	3(3.3%)	2(8.7%)	
> 20 mm	48(20.6%)	10(14.9%)	9(17.0%)	22(24.4%)	7(30.4%)	
Unknown	20(8.6%)	6(9.0%)	5(9.4%)	8(8.9%)	1(4.3%)	
*Venous invasion*						0.270
No	29(12.4%)	8(11.9%)	12(22.6%)	8(8.9%)	1(4.3%)	
Yes	41(17.6%)	12(17.9%)	8(15.1%)	17(18.9%)	4(17.4%)	
Unknown	163(70.0%)	47(70.1%)	33(62.3%)	65(72.2%)	18(78.3%)	
*Lymphatic invasion*						**0.031**
No	33(14.2%)	8(11.9%)	13(24.5%)	10(11.1%)	2(8.7%)	
Yes	60(25.8%)	23(34.3%)	5(9.4%)	25(27.8%)	7(30.4%)	
Unknown	140(60.1%)	36(53.7%)	35(66.0%)	55(61.1%)	14(60.9%)	
*Survival status*						**0.0001**
5y OS	34.8%	17.7%	57.4%	40.1%	6.8%	
10y OS	16.3%	7.9%	21.7%	22.3%	6.8%	
Median OS (months)	46	35	64	48	24	
Median 95%CI (months)	38.3–53.7	32.2–37.8	48.3–81.7	40.1–59.9	17.2–32.7	

Bold fonts represent *p* < 0.05

### Construction of the prognosis model and verification in validation data

**Fig. 4:**
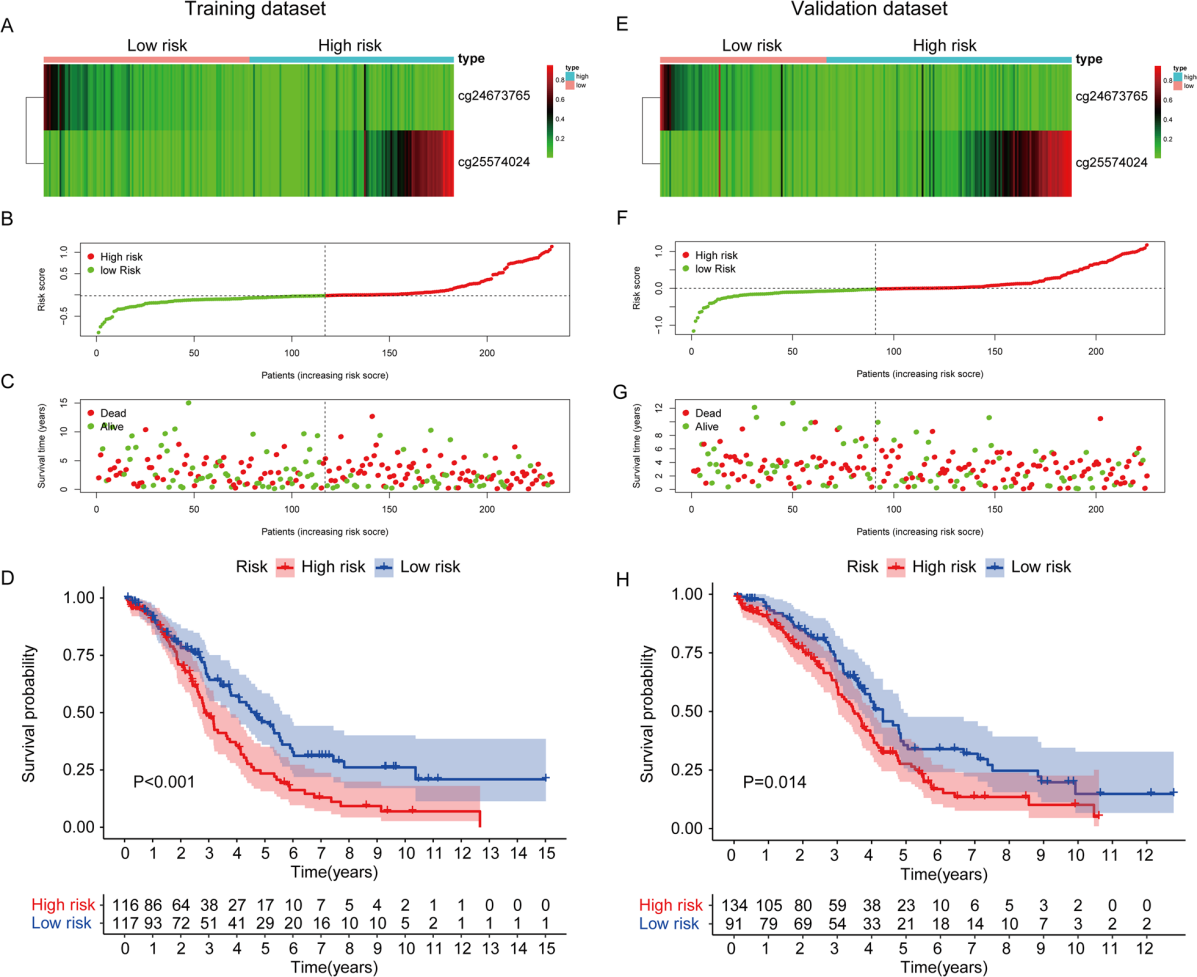
Construction and validation of the methylation-driven prognosis prediction model in HGSOC. **A** Consensus clustering of the two CpG sites in the training set. **B** Risk score distribution of HGSOC patients in the training data set. **C** Survival status of each HGSOC patient in the training data set. The risk score distribution is consistent with **B**. **D** Survival curves of two clusters predicted from the training set using the prognosis model. The log-rank test was used to assess the statistical significance of the difference (*p* < 0.01). **E** Consensus clustering of the two CpG sites in the validation set. **F** Risk score distribution of HGSOC patients in the validation data set. **G** Survival status of each HGSOC patient in the validation data set. The risk score distribution is consistent with **F**. **H** Survival curves of two clusters verified in the validation set using the prognosis model. The log-rank test was used to assess the statistical significance of the difference (*p* = 0.014)

Based on the four subtype classifications, we established a prognosis prediction model (Fig. 4). To optimize this model to contain only the most predictive genes, a stepwise Cox proportional hazards regression model was used, which identified two genes. Subsequently, a risk score was built: risk value = (1.207973 × cg25574024methylation value) − (1.35159 × cg24673765methylation value). The risk score for each patient was calculated using this formula. In the training cohort, patients were assigned into high-risk group and low-risk group based on the optimized risk value (Fig. 4A–C). Kaplan–Meier survival analyses showed that the rate of survival in the high-risk group was significantly higher than that in the low-risk group (Fig. 4D, *p* < 0.001). Furthermore, we used the validation cohort to validate the model. With the same formula, patients were divided into a high-risk group and low-risk group by their risk score (Fig. 4E–G). Additionally, results of OS showed that patients in the high-risk group had a significantly shorter survival than the counterparts (Fig. 4H, *p *= 0.014). Furthermore, the results of fivefold cross-validation show that the model established by the method in this paper has good performance in the prediction of prognosis, and the survival rate has significant differences between high-risk group and low-risk group in the fivefold cross-validation (Additional file 1: Fig. S1).

## Discussion

Classifications that are based solely on the pathological features of tissues have limitations. Remodelling of the epigenome is a fundamental factor in tumour prognosis prediction or risk stratification. DNA methylation is one of the most common epigenetic phenomena. It is universally acknowledged that DNA methylation alterations are potential molecular markers for cancer progression [30–33]. Several studies of ovarian cancer have reported that DNA methylation signatures play a pivotal role in the molecular classification, survival status, and adjuvant chemotherapy response [34–41]. Despite increasing knowledge of the most common type of ovarian cancer, HGSOC, there are no effective methylation-based molecular signatures [42]. Therefore, we performed the present study to better understand the extent and heterogeneity of aberrant DNA methylation in HGSOC.

The present study analysed prognosis-related methylation sites and obtained the corresponding signalling pathways, which provided the research directions for the methylation of HGSOC in future. We identified four methylation subgroups with different prognoses. There were divergent biological characteristics in the four methylation subgroups, which confirmed the heterogeneity of HGSOC and the necessity of cautious classification. Molecular-targeted therapy is gaining traction, and we may identify the underlying mechanisms in different methylation subgroups to help design new strategies in future. We further established a prognosis prediction model based on the significantly methylated sites using multivariate Cox analysis for the convenience of clinical application. Validation was performed to illustrate the reliability of this prediction model. This study identified stable classification with methylation patterns and clinical meaning, which is informative for HGSOC biological characteristics and prognosis.

Molecular mechanistic studies based on bioinformatics analyses are highly significant to cancer research. Previous studies used methylated DNA Immunoprecipitation in A2780 and CaOV3 ovarian cancer cell lines to confirm a panel of six gene promoters that differentiated serous EOC from normal ovarian surface epithelial cells [43]. Zeller et al. [44] identified loci at 4092 genes that were hypermethylated in chemoresistant A2780/cp70 cells compared to the parental-sensitive A2780 cell line. Further high-throughput DNA methylation profiling of 27 primary epithelial ovarian tumours and 15 ovarian cancer cell lines revealed significant differences in the DNA methylation profiles between ovarian cancer cell lines and tumours, which underscores the need for caution in the use of cell lines as tumour models for epigenetic molecular studies.

Based on the difference between ovarian tumours and cancer cell lines, preference is given to studies that directly examine the methylation profiles of cancer tissues. Notably, ovarian cancer is a heterogeneous disease that includes five major epithelial ovarian tumour subtypes (high- and low-grade serous, endometrioid, mucinous, and clear cell). Previous studies demonstrated that ovarian tumours of different histological types have distinct methylation profiles [45], which reinforces the need to treat different histotypes of ovarian cancer as separate diseases. For serous EOC, Keita et al. [34] found that widespread DNA hypermethylation occurs in tumours with low malignant potential (borderline) and significant DNA hypomethylation was observed only in grade 3 serous EOC tumours.

Although it is the most common histological type of EOC, the methylation status of HGSOC has not been studied in detail. Keita et al. [34] only included 10 cases of HGSOC, which could not be further studied as a separate group. Bodelon et al. [35] found three methylation subgroups in 61 HGSOCs. However, the small sample size is a limitation of the quality of data generated from these experiments, and the characteristics of methylation subgroups, which are a matter of cardinal importance for targeted therapy, were not elaborated in detail. Reyes et al. [46] focused on the difference in methylation between HGSOC and normal fallopian tube tissue and between primary and recurrent ovarian cancer. Montavon et al. [38] examined and compared the methylation patterns of 10 genes in a cohort of 80 primary HGSOC and 12 benign ovarian surface epithelium samples. Dai et al. [37] primarily profiled DNA methylation of genes in four pathways. Baranova et al. [39] identified the methylation of the CDH13, HNF1B, PCDH17, and GATA4 genes to distinguish HGSOC from normal samples. In summary, research on the methylation of HGSOC is limited to appointed methylation sites, differences between HGSOC and normal tissue, and differences between recurrent cancer and primary cancer. Previous TCGA studies [19] established a four-cluster system. However, the classification system was established based on multiple data integrations (mRNA and miRNA expression and DNA methylation) rather than single methylation data. The present study emphasized the importance of DNA methylation and considered DNA methylation as an independent system, which is different from TCGA articles.

Based on TCGA data, our study primarily focused on comprehensive methylation analyses of HGSOC. The corresponding genes of differentially methylated sites that were significant in the multivariate Cox analysis were analysed. Pathway enrichment analyses suggested that differentially methylated genes were primarily enriched in the MAPK signalling pathway, including the CACNB3, MAP3K12, PGF, MAPK13, and CSF1R genes. The MAPK signalling pathway plays a role in the regulation of gene expression, cellular growth, and survival, and it is implicated in most cancers. However, little research focused on the interaction between methylation modification and MAPK signalling pathway proteins. The glycolysis/gluconeogenesis pathway and pyruvate metabolism pathway were also enriched in KEGG analyses, and these pathways play a vital role in cancer progression [47–49]. However, the relationship between these metabolic pathways and DNA methylation is not known. DNA methylation may also be a direction in the study of proteoglycan pathways, including the VAV2, CCND1, DDX5, and ITPR2 genes.

The subtypes identified from the methylation profiling data classified HGSOC into four groups. Notably, the methylation levels of the different subgroups reflected different molecular features. C1 was associated with the hypomethylation of cg03848675, cg12493906, and cg13055001, which were annotated as FOXF2, MMP26, and PPP1CA, respectively. FOXF2 is a critical tumour suppressor in gastric carcinogenesis that mediates upregulation of the E3 ligase IRF2BPL to drive ubiquitylation and degradation of β-catenin, which blunts Wnt signalling and suppresses carcinogenesis [50]. The function of FOXC2 in breast cancer is inconsistent. Some researchers reported that FOXC2 suppressed epithelial–mesenchymal transition and multidrug resistance in basal-like breast cancer [51]. However, another study found that FOXF2 promoted the bone metastasis of breast cancer cells [52]. lncRNA ADAMTS9-AS2 decreased tumour progression in ovarian cancer by regulating the miR-182-5p/FOXF2 axis [53]. Matrix metalloproteases (MMPs) play a vital role in cancer metastasis. The immunostaining intensity of MMP-26 increased with ovarian tumour stage [54], which suggests a role for MMP-26 in ovarian cancer biological function. PPP1CA was associated with activation of the MAPK signalling pathway [55, 56]. Because the hypomethylation loci of the C1 subtype were closely related to tumour metastasis, C1 was defined as the metastasis subgroup. Targeted therapy inhibits metastasis may be more effective in this subgroup than the other subgroups.

The C2 subtype had the best prognosis and showed relative hypomethylation of cg13791131, cg25574024, cg24673765, and cg27239157, which were annotated as IGF2 (cg13791131, cg25574024), HSPB6 (cg24673765), and MCF2L2 (cg27239157), respectively. IGF2 plays a key role in glucose metabolism, HSPB6 might be associated with insulin resistance [27], and MCF2L2 might be one of the most important marker genes contributing to type 1 diabetes [28] and polycystic ovary syndrome [29]. All of these diseases are metabolic disorders, and these three genes are associated with metabolism. Therefore, C2 was defined as the metabolism subtype. Future studies will investigate whether this subtype is related to metabolic disorders and examine the application value of metabolic drugs in this subtype.

C3 presented with hypermethylation associated with cg03848675, which was opposite to the patterns observed in C1, and featured hypomethylation of cg14290451(RPL10A). RpL10A stimulates cell proliferation via the insulin signalling pathway [57]. C4 was the poorest prognostic group and exhibited hypermethylation at 54 methylation loci. The hypermethylation of tumour suppressor genes contributes to a more aggressive phenotype. Therefore, C4 was defined as the hypermethylation subtype, which suggests that demethylation agents could be preclinically tested for this group. It will be of great interest to clarify the underlying reasons for these unique subtypes and elucidate the relationship between different subtypes and their level of sensitivity to specific targeted agents. However, care must be taken. Intervening therapeutically to reverse a pattern seen in a cluster may have adverse effects. The adverse effects of therapeutic drugs require structural transformation by pharmaceutics, but this process is a long way off.

Clinical features, including survival outcome, residual tumours, and lymphatic invasion, were markedly different between the four subgroups. The metabolism subtype was associated with no macroscopic residual tumour and a high probability of negative lymphatic invasion status, which could explain the favourable prognosis. Notably, the frequency of residual tumours in the hypermethylation subtype was higher than that in the other subtypes, which indicates that this group could be treated with neoadjuvant chemotherapy to improve the quality of surgery and reduce the possibility of residual lesions.

We further developed a prediction model for prognostication and clinical application. The prognostic model distinguished the training data sets and the validation sets into different prognosis clusters. This clinically promising model may be used to predict the prognosis of HGSOC patients, and follow-up may be strengthened for high-risk patients.

## Conclusion

The results of this exploratory study suggest four distinct HGSOC clusters that are distinguishable with DNA methylation profiling and highlight several important genetic characteristics. We showed that the metabolism subtype had a favourable prognosis and that the hypermethylation subtype had the worst prognosis. This result provides a more detailed explanation of HGSOC heterogeneity. Due to the high rates of residual sites, hypermethylation subtype tumours may be treated with neoadjuvant chemotherapy to improve the quality of surgery and reduce the residual rate. The sensitivity to demethylation agents in this subtype should be examined and elucidated. Agents regulating metabolism may be effective for the metabolism subtype, and agents disrupting tumour metastasis may have value for further exploration in the metastasis subtype. Our prediction model provides guidance for clinicians in decisions related to prognosis. Our findings lay the groundwork for an improved understanding of the methylation-based subtypes of HGSOC and provide a useful resource with clinical implications for further studies.

## Supplementary Information

**Additional file 1: Fig. S1.** The results of fivefold cross-validation in patients of training group.

**Additional file 2: Table S1.** A multivariate Cox proportional hazard regression model revealed that 780 sites were significantly related to survival.

**Additional file 3: Table S2.** The top 20 statistically significant biological processes based on corresponding genes derived from the differentially methylated probe (Table S1)

**Additional file 4: Table S3.** The top 20 statistically significant molecular functions based on corresponding genes derived from the differentially methylated probe (Table S1).

**Additional file 5: Table S4.** The top 20 statistically significant pathways according to Kyoto Encyclopedia of Genes and Genomes pathway analysis(Table S1).

**Additional file 6: Table S5.** The methylation loci and corresponding genes involved in MAPK signalling, pyruvate metabolism, glycolysis, and proteoglycan pathways.

**Additional file 7: Table S6.** The molecular characteristics of cluster 1 to cluster 4.

## Data Availability

Clinical information and DNA methylation data were retrieved from the TCGA data portal, which is a publicly available database.

## Abbreviations

EOC: Epithelial ovarian cancer
HGSOC: High-grade serous ovarian cancer
TCGA: The Cancer Genome Atlas
CpGs: Cytosines preceding guanosine sites
GO: Gene ontology
KEGG: Kyoto Encyclopedia of Genes and Genomes
MAPK: Mitogen-activated protein kinase
