# Supplementary figures and images for: DNA methylation-based profiling reveals distinct clusters with survival heterogeneity in high-grade serous ovarian cancer
Source: Clin Epigenetics. 2021 Oct 13;13:190. doi: 10.1186/s13148-021-01178-3 (PMC8515755; doi:10.1186/s13148-021-01178-3)

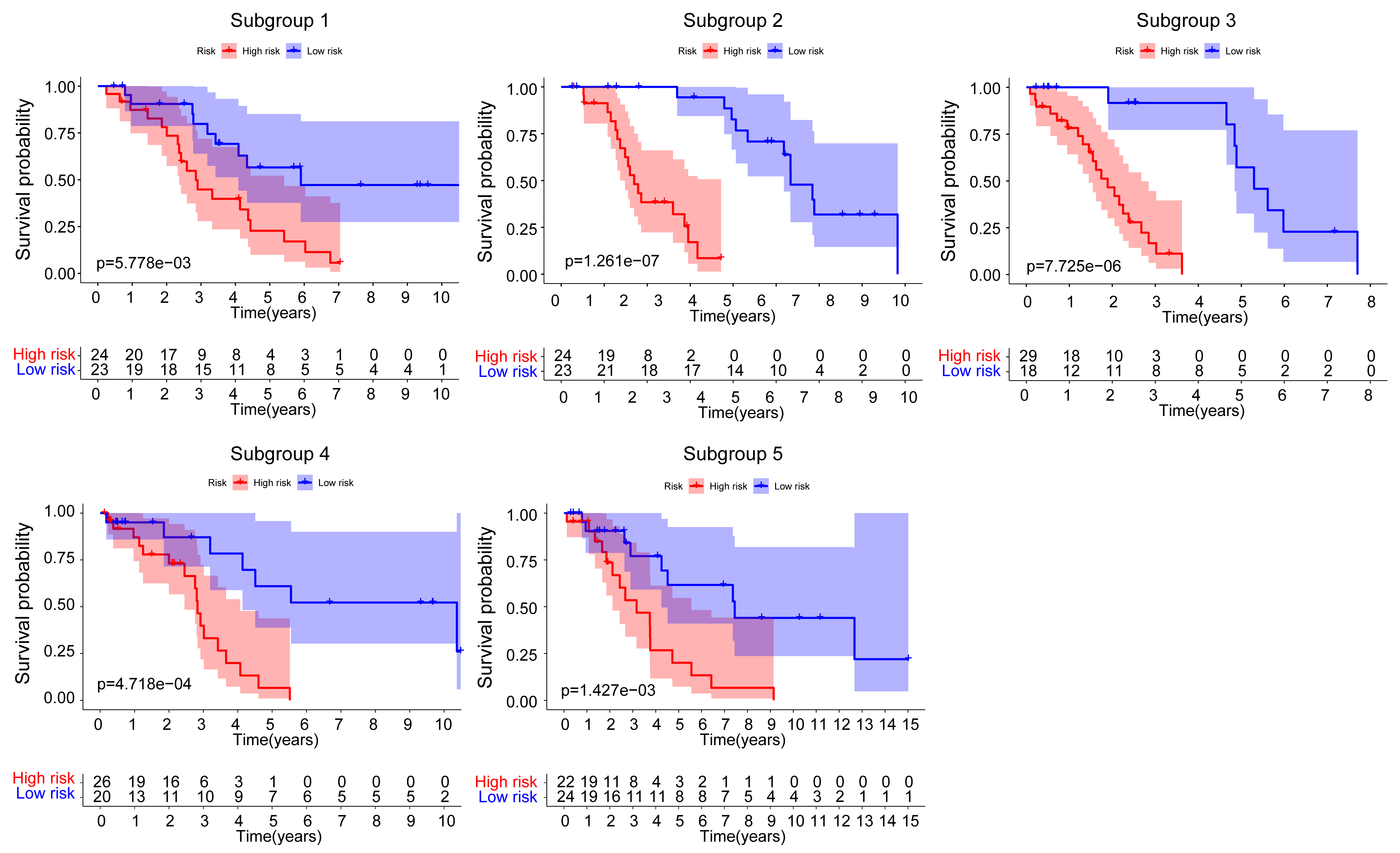

Supplement: Supplementary file 1 — Additional file 1: Fig. S1. The results of fivefold cross-validation in patients of training group. [file 13148_2021_1178_MOESM1_ESM.tif]
