# Supplementary material for: DNA methylation-based profiling reveals distinct clusters with survival heterogeneity in high-grade serous ovarian cancer
Source: Clin Epigenetics. 2021 Oct 13;13:190. doi: 10.1186/s13148-021-01178-3 (PMC8515755; doi:10.1186/s13148-021-01178-3)
Supplement: Supplementary file 3 — Additional file 3: Table S2. The top 20 statistically significant biological processes based on corresponding genes derived from the differentially methylated probe (Table S1) [file 13148_2021_1178_MOESM3_ESM.docx]

**Table 2.** The top 20 statistically significant biological process based on corresponding genes derived from the differentially methylated probe ([supplement table 1](https://www.ncbi.nlm.nih.gov/pmc/articles/PMC5530656/#d35e1031))

| ID | Description | GeneRatio | BgRatio | pvalue |
| --- | --- | --- | --- | --- |
| GO:0043543 | protein acylation | 22/734 | 248/18670 | 0.000334 |
| GO:0015980 | energy derivation by oxidation of organic compounds | 23/734 | 285/18670 | 0.000929 |
| GO:0008380 | RNA splicing | 33/734 | 469/18670 | 0.000949 |
| GO:0006605 | protein targeting | 31/734 | 432/18670 | 0.000976 |
| GO:0000377 | RNA splicing, via transesterification reactions with bulged adenosine as nucleophile | 28/734 | 379/18670 | 0.001091 |
| GO:0000398 | mRNA splicing, via spliceosome | 28/734 | 379/18670 | 0.001091 |
| GO:0006622 | protein targeting to lysosome | 5/734 | 21/18670 | 0.001115 |
| GO:0000375 | RNA splicing, via transesterification reactions | 28/734 | 382/18670 | 0.001228 |
| GO:0044090 | positive regulation of vacuole organization | 4/734 | 14/18670 | 0.00173 |
| GO:0016072 | rRNA metabolic process | 20/734 | 253/18670 | 0.002475 |
| GO:0046677 | response to antibiotic | 24/734 | 327/18670 | 0.002611 |
| GO:0000082 | G1/S transition of mitotic cell cycle | 21/734 | 279/18670 | 0.003504 |
| GO:1902175 | regulation of oxidative stress-induced intrinsic apoptotic signaling pathway | 5/734 | 27/18670 | 0.003641 |
| GO:0016246 | RNA interference | 4/734 | 17/18670 | 0.003744 |
| GO:0018904 | ether metabolic process | 4/734 | 17/18670 | 0.003744 |
| GO:0007599 | hemostasis | 24/734 | 341/18670 | 0.004436 |
| GO:0043331 | response to dsRNA | 7/734 | 53/18670 | 0.004508 |
| GO:0051023 | regulation of immunoglobulin secretion | 4/734 | 18/18670 | 0.004666 |
| GO:0031667 | response to nutrient levels | 32/734 | 499/18670 | 0.004734 |
| GO:0043255 | regulation of carbohydrate biosynthetic process | 10/734 | 97/18670 | 0.004796 |
