# Supplementary material for: DNA methylation-based profiling reveals distinct clusters with survival heterogeneity in high-grade serous ovarian cancer
Source: Clin Epigenetics. 2021 Oct 13;13:190. doi: 10.1186/s13148-021-01178-3 (PMC8515755; doi:10.1186/s13148-021-01178-3)
Supplement: Supplementary file 4 — Additional file 4: Table S3. The top 20 statistically significant molecular functions based on corresponding genes derived from the differentially methylated probe (Table S1). [file 13148_2021_1178_MOESM4_ESM.docx]

**Table 4.** The top 20 statistically significant molecular function based on corresponding genes derived from the differentially methylated probe ([supplement table 1](https://www.ncbi.nlm.nih.gov/pmc/articles/PMC5530656/#d35e1031)).

| ID | Description | GeneRatio | BgRatio | pvalue |
| --- | --- | --- | --- | --- |
| GO:0003725 | double-stranded RNA binding | 10/735 | 75/17697 | 0.001037 |
| GO:0004707 | MAP kinase activity | 4/735 | 14/17697 | 0.002116 |
| GO:0051087 | chaperone binding | 11/735 | 102/17697 | 0.00338 |
| GO:0030145 | manganese ion binding | 8/735 | 62/17697 | 0.00397 |
| GO:0009055 | electron transfer activity | 11/735 | 114/17697 | 0.007822 |
| GO:0008083 | growth factor activity | 14/735 | 163/17697 | 0.008101 |
| GO:0042393 | histone binding | 16/735 | 197/17697 | 0.008198 |
| GO:0016679 | oxidoreductase activity, acting on diphenols and related substances as donors | 3/735 | 11/17697 | 0.009168 |
| GO:0099106 | ion channel regulator activity | 11/735 | 118/17697 | 0.010033 |
| GO:0004298 | threonine-type endopeptidase activity | 4/735 | 21/17697 | 0.010045 |
| GO:0070003 | threonine-type peptidase activity | 4/735 | 21/17697 | 0.010045 |
| GO:0005041 | low-density lipoprotein particle receptor activity | 3/735 | 12/17697 | 0.011851 |
| GO:0031543 | peptidyl-proline dioxygenase activity | 3/735 | 12/17697 | 0.011851 |
| GO:0048029 | monosaccharide binding | 8/735 | 75/17697 | 0.012401 |
| GO:0016860 | intramolecular oxidoreductase activity | 6/735 | 47/17697 | 0.012667 |
| GO:0017017 | MAP kinase tyrosine/serine/threonine phosphatase activity | 3/735 | 13/17697 | 0.014937 |
| GO:0043394 | proteoglycan binding | 5/735 | 36/17697 | 0.015814 |
| GO:0016247 | channel regulator activity | 12/735 | 144/17697 | 0.016952 |
| GO:0016863 | intramolecular oxidoreductase activity, transposing C=C bonds | 3/735 | 14/17697 | 0.018434 |
| GO:0098847 | sequence-specific single stranded DNA binding | 3/735 | 14/17697 | 0.018434 |
