# Supplementary material for: DNA methylation-based profiling reveals distinct clusters with survival heterogeneity in high-grade serous ovarian cancer
Source: Clin Epigenetics. 2021 Oct 13;13:190. doi: 10.1186/s13148-021-01178-3 (PMC8515755; doi:10.1186/s13148-021-01178-3)
Supplement: Supplementary file 5 — Additional file 5: Table S4. The top 20 statistically significant pathways according to Kyoto Encyclopedia of Genes and Genomes pathway analysis(Table S1). [file 13148_2021_1178_MOESM5_ESM.docx]

**Table 5.** The top 20 statistically significant pathways analyzed by Kyoto Encyclopedia of Genes and Genomes pathway analysis.

| ID | Description | GeneRatio | BgRatio | *P*-value |
| --- | --- | --- | --- | --- |
| hsa04010 | MAPK signaling pathway | 23/352 | 294/8048 | 0.004805 |
| hsa05014 | Amyotrophic lateral sclerosis | 27/352 | 364/8048 | 0.004845 |
| hsa04261 | Adrenergic signaling in cardiomyocytes | 14/352 | 149/8048 | 0.005544 |
| hsa03040 | Spliceosome | 14/352 | 150/8048 | 0.005879 |
| hsa04714 | Thermogenesis | 19/352 | 231/8048 | 0.005918 |
| hsa00620 | Pyruvate metabolism | 6/352 | 39/8048 | 0.006442 |
| hsa05016 | Huntington disease | 23/352 | 306/8048 | 0.007732 |
| hsa05205 | Proteoglycans in cancer | 17/352 | 205/8048 | 0.008309 |
| hsa00010 | Glycolysis / Gluconeogenesis | 8/352 | 67/8048 | 0.008468 |
| hsa00380 | Tryptophan metabolism | 6/352 | 42/8048 | 0.009282 |
| hsa04070 | Phosphatidylinositol signaling system | 10/352 | 97/8048 | 0.009615 |
| hsa05120 | Epithelial cell signaling in Helicobacter pylori infection | 8/352 | 70/8048 | 0.010949 |
| hsa00230 | Purine metabolism | 12/352 | 130/8048 | 0.011291 |
| hsa04625 | C-type lectin receptor signaling pathway | 10/352 | 104/8048 | 0.015269 |
| hsa00310 | Lysine degradation | 7/352 | 61/8048 | 0.016513 |
| hsa04912 | GnRH signaling pathway | 9/352 | 93/8048 | 0.020048 |
| hsa04114 | Oocyte meiosis | 11/352 | 128/8048 | 0.024191 |
| hsa05132 | Salmonella infection | 16/352 | 213/8048 | 0.024228 |
| hsa05012 | Parkinson disease | 18/352 | 249/8048 | 0.024714 |
| hsa01522 | Endocrine resistance | 9/352 | 98/8048 | 0.027166 |
