# Supplementary material for: DNA methylation-based profiling reveals distinct clusters with survival heterogeneity in high-grade serous ovarian cancer
Source: Clin Epigenetics. 2021 Oct 13;13:190. doi: 10.1186/s13148-021-01178-3 (PMC8515755; doi:10.1186/s13148-021-01178-3)
Supplement: Supplementary file 7 — Additional file 7: Table S6. The molecular characteristics of cluster 1 to cluster 4. [file 13148_2021_1178_MOESM7_ESM.docx]

**Table S6.** The molecular characteristics of cluster1 to cluster 4.

|  | **Composite Element REF** | **β-value** | **Chromosome** | **Gene_Symbol;Gene_Type** | **Function** | **Feature Type** |
| --- | --- | --- | --- | --- | --- | --- |
| C1  metastasis | cg03848675 | 0.153267 | chr6 | FOXF2 | Ubiquitination and degradation of CTNNB1[1], tumor metastasis[2,3] | Island |
|  | cg12493906 | 0.386715 | chr11 | MMP26; | Tumor invasion and metastasis[4] | . |
|  | cg13055001 | 0.119741 | chr11 | PPP1CA; | Associated with MAPK[5,6] | S_Shore |
| C2  metabolism | cg13791131；  cg25574024 | 0.437158；  0.522035 | chr11 | IGF2; IGF2-AS; INS-IGF2 | Glucose metabolism; | Island |
|  | cg24673765 | 0.149446 | chr19 | HSPB6; PROSER3; | Associated with insulin resistance [7] | Island |
|  | cg27239157 | 0.080535 | chr3 | MCF2L2; | Polycystic ovary syndrome and type 1 Diabetes [8,9] | Island |
| C3 | cg14290451 | 0.032448 | chr6 | RPL10A; | Stimulates cell proliferation via the insulin signaling pathway[10] | Island |
|  | cg03848675 | 0.153267 | chr6 | FOXF2 | Ubiquitination and degradation of CTNNB1[1], tumor metastasis[2,3] | Island |
| C4  hypermethylation | cg27239157 | 0.080535 | chr3 | MCF2L2; | Polycystic ovary syndrome and type 1 Diabetes [8,9] | Island |
|  | cg13791131；  cg25574024 | 0.437158；  0.522035 | chr11 | IGF2; IGF2-AS; INS-IGF2 | Glucose metabolism; | Island |
|  | cg14290451 | 0.032448 | chr6 | RPL10A; | Stimulates cell proliferation via the insulin signaling pathway[10] | Island |
|  | cg00123035 | 0.068367 | chr1 | RFX5; RP11-126K1.6 | MHC-II promoters[11,12]; | Island |
|  | cg00420715 | 0.038548 | chr8 | RP11-410L14.2; VPS13B | Cohen syndrome[13,14] | N_Shore |
|  | cg01468868 | 0.077478 | chr22 | LIF; RP1-102K2.8 | Pancreatic cancer[15], PD-1 [16,17] [18], cancer dissemination and local invasion[19], ovarian cancer[20] | Island |
|  | cg01968530 | 0.025832 | chr18 | TMEM241; | Carbohydrate transport（GO:0008643） | Island |
|  | cg02868338 | 0.059588 | chr8 | CTD-3065J16.9; EXOSC4; | Positive regulation of cell growth[21] | N_Shore |
|  | cg03027037 | 0.023587 | chr20 | NAPB; | Golgi-to-ER retrograde transport | S_Shore |
|  | cg03849074 | 0.094198 | chr6 | ZKSCAN3; ZSCAN31; | Negative regulation of autophagy[22],promotes cancer cell progression and/or migration in various tumors and myelomas[23,24,22,25,26] | . |
|  | cg04305134 | 0.103203 | chr10 | ANXA7; | Anticancer function[27-31]： | N_Shore |
|  | cg05722918 | 0.090458 | chr12 | SLC5A8 | Chain fatty acids, a tumor suppressor[32-35], histone acetylation[36] | Island |
|  | cg06797533 | 0.025623 | chr9 | PLIN2; | Metabolism of lipids and lipoproteins[37,38], HIF-1-alpha transcription factor network[39,40] , a tumor suppressor [41-43] | Island |
|  | cg06911113 | 0.051303 | chr1 | UBXN10; UBXN10-AS1 | Cilia formation | Island |
|  | cg07038887 | 0.017678 | chr17 | BLMH; | a tumor suppressor [44], Drug sensitivity [45,46] | Island |
|  | cg07190917 | 0.053745 | chr16 | STX4; | Stabilization and expansion of the E-cadherin adherens junction[47], a tumor suppressor [48], Low expression promotes autophagy [49], Facilitating transfer[50], Insulin-mediated glucose transport, MT1-MMP[47] | Island |
|  | cg07740989 | 0.043114 | chr6 | EEF1A1; | Cellular response to epidermal growth factor stimulus[51][52-56,51] | Island |
|  | cg08376864 | 0.183557 | chr3 | HRASLS | MAPK cascade, Hypermethylation in tumors [57,58], | Island |
|  | cg09439093 | 0.033929 | chr6 | ECI2; RP3-400B16.1; RP3-400B16.4 | Fatty acid beta-oxidation (GO:0006635) | Island |
|  | cg09593402 | 0.092498 | chr5 | CTD-2260A17.2;LNPEP; | Adaptive Immune System; Class I MHC mediated antigen processing and presentation | N_Shore |
|  | cg09659887 | 0.039208 | chr11 | IPO7; | Promote proliferation, invasion and migration [59-62] | Island |
|  | cg09801842 | 0.01491 | chr5 | SFXN1; | Ion transport[63], One carbon metabolism [64], It is associated with poor prognosis of lung adenocarcinoma [65] | Island |
|  | cg10985987 | 0.047634 | chr16 | CDIPT;CDIPT-AS1;CTD-2574D22.7 | None related research | Island |
|  | cg11868900 | 0.275769 | chr8 | EFCAB1; | Tumor suppressor [66,67] | S_Shore |
|  | cg12316147 | 0.031216 | chr9 | GBA2; RGP1; | Tumor suppressor [68,69], lipid metabolic process | Island |
|  | cg12352006 | 0.038934 | chr22 | UQCR10; ZMAT5; | Mitochondrial respiratory chain complex III assembly[70][71] | S_Shore |
|  | cg12385425 | 0.05569 | chr2 | TMEM17; | The relationship with tumor is unclear[72-74] | S_Shore |
|  | cg13912117 | 0.739511 | chr8 | ADCY8 | Tumor suppressor [75,76], Tumor suppressor [77] | Island |
|  | cg15401952 | 0.051192 | chr4 | KCNIP4;RP11-120A1.1 | Tumor suppressor [79-81] | . |
|  | cg16108132 | 0.047614 | chr10 | ANXA7; | None related research | S_Shore |
|  | cg16178491 | 0.080079 | chr12 | SLC5A8 | None related research | S_Shore |
|  | cg16267059 | 0.056157 | chr15 | MFAP1 | It's not about the tumor [82] | S_Shore |
|  | cg16545079 | 0.079649 | chr17 | PER1;RP11-599B13.6 | Tumor suppressor [83-86] | S_Shore |
|  | cg17057965 | 0.021663 | chr1 | FBXO28; | Cancer promoting [87] | N_Shore |
|  | cg17746675 | 0.039912 | chr3 | TAMM41; | lipid metabolic process | Island |
|  | cg17820591 | 0.037709 | chr17 | ENO3;PFN1; | Tumor suppressor [89], glycolytic process (GO:0006096), Cancer promoting [90,91], Tumor suppressor [92], Immune related [94] | Island |
|  | cg17868994 | 0.057781 | chr9 | NIPSNAP3B; | None related research | Island |
|  | cg19461621 | 0.501833 | chr18 | COLEC12; | immune response[95], Estrogen receptors (ERs) primary target genes[96] | Island |
|  | cg19747852 | 0.01018 | chr7 | TRA2A; | Cancer promoting [97-99], histone methylation [100,101], RNA methylation [102], neuroblastoma therapeutic effects[103] | Island |
|  | cg19921353 | 0.114097 | chr6 | HLA-DPA1; HLA-DPB1; | Immune system process (GO:0002376), antigen processing and presentation (GO:0019882), T cell receptor signaling pathway (GO:0050852) | . |
|  | cg20092036 | 0.350285 | chr12 | KCTD10;UBE3B; | ubiquitin-dependent protein catabolic process (GO:0006511)[104-107] | N_Shore |
|  | cg21184495 | 0.175639 | chr17 | C17orf75;MIR632;RP11-227G15.3;ZNF207; | Promoting tumor growth and drug resistance [108-110] | Island |
|  | cg21238457 | 0.063033 | chr11 | WNT11; | Beta-catenin independent WNT signaling; [111,112] | Island |
|  | cg21833459 | 0.047533 | chr11 | FAM111B; | DNA replication (GO:0006260)[113], Cancer promoting[114] | Island |
|  | cg22367989 | 0.020182 | chr2 | NRP2; | Angiogenesis(GO:0001525[115,116]), Cisplatin resistance [117], Cancer promoting[118,119] | Island |
|  | cg22598028 | 0.024412 | chr3 | RP11-944L7.4;RP11-944L7.5;ZNF197;ZNF660 | Copper metabolism and placentation[120], tumor related [121] | Island |
|  | cg23347958 | 0.07726 | chr17 | DHX8; | A tandem duplication of BRCA1 exons 1-19 through DHX8 exon 2 in four families with hereditary breast and ovarian cancer syndrome | Island |
|  | cg24028122 | 0.058413 | chr6 | UFL1; UFL1-AS1 | Positive regulation of cell proliferation[122], negative regulation of NF-kappaB transcription factor activity[123], negative regulation of protein ubiquitination(GO:0031397)[124-127] | Island |
|  | cg24997562 | 0.102809 | chr12 | OASL; | Immune system process (GO:0002376；[128]), drug-sensitivity to cisplatin [129]； | . |
|  | cg25123470 | 0.084771 | chr10 | KCNIP2; | Ion transport (GO:0006811)[130] | S_Shore |
|  | cg25903497 | 0.017237 | chr2 | PNKD;TMBIM1; | Negative regulation of neurotransmitter secretion(GO:0046929), Tumor related [131] | Island |
|  | cg26239051 | 0.032487 | chr2 | EHBP1 | Actin cytoskeleton organization（GO:0030036；[132] | Island |

1. Higashimori A, Dong Y, Zhang Y, Kang W, Nakatsu G, Ng SSM, Arakawa T, Sung JJY, Chan FKL, Yu J (2018) Forkhead Box F2 Suppresses Gastric Cancer through a Novel FOXF2-IRF2BPL-β-Catenin Signaling Axis. Cancer research 78 (7):1643-1656. doi:10.1158/0008-5472.Can-17-2403

2. Cai J, Tian AX, Wang QS, Kong PZ, Du X, Li XQ, Feng YM (2015) FOXF2 suppresses the FOXC2-mediated epithelial-mesenchymal transition and multidrug resistance of basal-like breast cancer. Cancer letters 367 (2):129-137. doi:10.1016/j.canlet.2015.07.001

3. Wang S, Li GX, Tan CC, He R, Kang LJ, Lu JT, Li XQ, Wang QS, Liu PF, Zhai QL, Feng YM (2019) FOXF2 reprograms breast cancer cells into bone metastasis seeds. Nature communications 10 (1):2707. doi:10.1038/s41467-019-10379-7

4. Scheau C, Badarau IA, Costache R, Caruntu C, Mihai GL, Didilescu AC, Constantin C, Neagu M (2019) The Role of Matrix Metalloproteinases in the Epithelial-Mesenchymal Transition of Hepatocellular Carcinoma. Analytical cellular pathology (Amsterdam) 2019:9423907. doi:10.1155/2019/9423907

5. Chen M, Wan L, Zhang J, Zhang J, Mendez L, Clohessy JG, Berry K, Victor J, Yin Q, Zhu Y, Wei W, Pandolfi PP (2018) Deregulated PP1α phosphatase activity towards MAPK activation is antagonized by a tumor suppressive failsafe mechanism. Nature communications 9 (1):159. doi:10.1038/s41467-017-02272-y

6. Sun H, Ou B, Zhao S, Liu X, Song L, Liu X, Wang R, Peng Z (2019) USP11 promotes growth and metastasis of colorectal cancer via PPP1CA-mediated activation of ERK/MAPK signaling pathway. EBioMedicine 48:236-247. doi:10.1016/j.ebiom.2019.08.061

7. Wang Y, Xu A, Ye J, Kraegen EW, Tse CA, Cooper GJ (2001) Alteration in phosphorylation of P20 is associated with insulin resistance. Diabetes 50 (8):1821-1827. doi:10.2337/diabetes.50.8.1821

8. Zhang D, Efendic S, Brismar K, Gu HF (2010) Effects of MCF2L2, ADIPOQ and SOX2 genetic polymorphisms on the development of nephropathy in type 1 Diabetes Mellitus. BMC medical genetics 11:116. doi:10.1186/1471-2350-11-116

9. Zheng Q, Shi Y, Yang Z, Xu X, Wang L, Xue F, Gu HF, Chen ZJ (2009) Family-based association study of the MCF2L2 gene and polycystic ovary syndrome. Gynecologic and obstetric investigation 68 (3):171-173. doi:10.1159/000231520

10. Chaichanit N, Wonglapsuwan M, Chotigeat W (2018) Ribosomal protein L10A and signaling pathway. Gene 674:170-177. doi:10.1016/j.gene.2018.06.081

11. Sugiaman-Trapman D, Vitezic M, Jouhilahti EM, Mathelier A, Lauter G, Misra S, Daub CO, Kere J, Swoboda P (2018) Characterization of the human RFX transcription factor family by regulatory and target gene analysis. BMC genomics 19 (1):181. doi:10.1186/s12864-018-4564-6

12. Yavorski JM, Blanck G (2017) MHC class II associated stomach cancer mutations correlate with lack of subsequent tumor development. Mol Clin Oncol 7 (6):1119-1121. doi:10.3892/mco.2017.1432

13. Abe A, Yamamoto Y, Katsumi A, Okamoto A, Tokuda M, Inaguma Y, Yamamoto K, Yanada M, Kanie T, Tomita A, Akatsuka Y, Okamoto M, Kameyama T, Mayeda A, Emi N (2018) Rearrangement of VPS13B, a causative gene of Cohen syndrome, in a case of RUNX1-RUNX1T1 leukemia with t(8;12;21). International journal of hematology 108 (2):208-212. doi:10.1007/s12185-017-2387-x

14. Momtazmanesh S, Rayzan E, Shahkarami S, Rohlfs M, Klein C, Rezaei N (2020) A novel VPS13B mutation in Cohen syndrome: a case report and review of literature. BMC medical genetics 21 (1):140. doi:10.1186/s12881-020-01075-1

15. Shi Y, Gao W, Lytle NK, Huang P, Yuan X, Dann AM, Ridinger-Saison M, DelGiorno KE, Antal CE, Liang G, Atkins AR, Erikson G, Sun H, Meisenhelder J, Terenziani E, Woo G, Fang L, Santisakultarm TP, Manor U, Xu R, Becerra CR, Borazanci E, Von Hoff DD, Grandgenett PM, Hollingsworth MA, Leblanc M, Umetsu SE, Collisson EA, Scadeng M, Lowy AM, Donahue TR, Reya T, Downes M, Evans RM, Wahl GM, Pawson T, Tian R, Hunter T (2019) Targeting LIF-mediated paracrine interaction for pancreatic cancer therapy and monitoring. Nature 569 (7754):131-135. doi:10.1038/s41586-019-1130-6

16. Vazquez JM, Sulak M, Chigurupati S, Lynch VJ (2018) A Zombie LIF Gene in Elephants Is Upregulated by TP53 to Induce Apoptosis in Response to DNA Damage. Cell reports 24 (7):1765-1776. doi:10.1016/j.celrep.2018.07.042

17. Pascual-García M, Bonfill-Teixidor E, Planas-Rigol E, Rubio-Perez C, Iurlaro R, Arias A, Cuartas I, Sala-Hojman A, Escudero L, Martínez-Ricarte F, Huber-Ruano I, Nuciforo P, Pedrosa L, Marques C, Braña I, Garralda E, Vieito M, Squatrito M, Pineda E, Graus F, Espejo C, Sahuquillo J, Tabernero J, Seoane J (2019) LIF regulates CXCL9 in tumor-associated macrophages and prevents CD8(+) T cell tumor-infiltration impairing anti-PD1 therapy. Nature communications 10 (1):2416. doi:10.1038/s41467-019-10369-9

18. Welc SS, Flores I, Wehling-Henricks M, Ramos J, Wang Y, Bertoni C, Tidball JG (2019) Targeting a therapeutic LIF transgene to muscle via the immune system ameliorates muscular dystrophy. Nature communications 10 (1):2788. doi:10.1038/s41467-019-10614-1

19. Liu SC, Hsu T, Chang YS, Chung AK, Jiang SS, OuYang CN, Yuh CH, Hsueh C, Liu YP, Tsang NM (2018) Cytoplasmic LIF reprograms invasive mode to enhance NPC dissemination through modulating YAP1-FAK/PXN signaling. Nature communications 9 (1):5105. doi:10.1038/s41467-018-07660-6

20. McLean K, Tan L, Bolland DE, Coffman LG, Peterson LF, Talpaz M, Neamati N, Buckanovich RJ (2019) Leukemia inhibitory factor functions in parallel with interleukin-6 to promote ovarian cancer growth. Oncogene 38 (9):1576-1584. doi:10.1038/s41388-018-0523-6

21. Pan Y, Tong JHM, Kang W, Lung RWM, Chak WP, Chung LY, Wu F, Li H, Yu J, Chan AWH, To KF (2018) EXOSC4 functions as a potential oncogene in development and progression of colorectal cancer. Molecular carcinogenesis 57 (12):1780-1791. doi:10.1002/mc.22896

22. Chauhan S, Goodwin JG, Chauhan S, Manyam G, Wang J, Kamat AM, Boyd DD (2013) ZKSCAN3 is a master transcriptional repressor of autophagy. Mol Cell 50 (1):16-28. doi:10.1016/j.molcel.2013.01.024

23. Lee S, Cho YE, Kim JY, Park JH (2018) ZKSCAN3 Upregulation and Its Poor Clinical Outcome in Uterine Cervical Cancer. International journal of molecular sciences 19 (10). doi:10.3390/ijms19102859

24. Chi Y, Xu H, Wang F, Chen X, Shan Z, Sun Y, Fan Q (2018) ZKSCAN3 promotes breast cancer cell proliferation, migration and invasion. Biochemical and biophysical research communications 503 (4):2583-2589. doi:10.1016/j.bbrc.2018.07.019

25. Kim CW, Roh SA, Tak KH, Koh BM, Ha YJ, Cho DH, Kim SY, Kim YS, Kim JC (2016) ZKSCAN3 Facilitates Liver Metastasis of Colorectal Cancer Associated with CEA-expressing Tumor. Anticancer research 36 (5):2397-2406

26. Yang L, Hamilton SR, Sood A, Kuwai T, Ellis L, Sanguino A, Lopez-Berestein G, Boyd DD (2008) The previously undescribed ZKSCAN3 (ZNF306) is a novel "driver" of colorectal cancer progression. Cancer research 68 (11):4321-4330. doi:10.1158/0008-5472.Can-08-0407

27. Hsu PI, Huang MS, Chen HC, Hsu PN, Lai TC, Wang JL, Lo GH, Lai KH, Tseng CJ, Hsiao M (2008) The significance of ANXA7 expression and its correlation with poor cellular differentiation and enhanced metastatic potential of gastric cancer. Journal of surgical oncology 97 (7):609-614. doi:10.1002/jso.21046

28. Leighton X, Eidelman O, Jozwik C, Pollard HB, Srivastava M (2017) ANXA7-GTPase as Tumor Suppressor: Mechanisms and Therapeutic Opportunities. Methods Mol Biol 1513:23-35. doi:10.1007/978-1-4939-6539-7_3

29. Liu S, Li X, Lin Z, Su L, Yan S, Zhao B, Miao J (2018) SEC-induced activation of ANXA7 GTPase suppresses prostate cancer metastasis. Cancer letters 416:11-23. doi:10.1016/j.canlet.2017.12.008

30. Srivastava M, Torosyan Y, Raffeld M, Eidelman O, Pollard HB, Bubendorf L (2007) ANXA7 expression represents hormone-relevant tumor suppression in different cancers. International journal of cancer Journal international du cancer 121 (12):2628-2636. doi:10.1002/ijc.23008

31. Torosyan Y, Dobi A, Glasman M, Mezhevaya K, Naga S, Huang W, Paweletz C, Leighton X, Pollard HB, Srivastava M (2010) Role of multi-hnRNP nuclear complex in regulation of tumor suppressor ANXA7 in prostate cancer cells. Oncogene 29 (17):2457-2466. doi:10.1038/onc.2010.2

32. Li H, Myeroff L, Smiraglia D, Romero MF, Pretlow TP, Kasturi L, Lutterbaugh J, Rerko RM, Casey G, Issa JP, Willis J, Willson JK, Plass C, Markowitz SD (2003) SLC5A8, a sodium transporter, is a tumor suppressor gene silenced by methylation in human colon aberrant crypt foci and cancers. Proceedings of the National Academy of Sciences of the United States of America 100 (14):8412-8417. doi:10.1073/pnas.1430846100

33. Helm J, Coppola D, Ganapathy V, Lloyd M, Centeno BA, Chen DT, Malafa MP, Park JY (2012) SLC5A8 nuclear translocation and loss of expression are associated with poor outcome in pancreatic ductal adenocarcinoma. Pancreas 41 (6):904-909. doi:10.1097/MPA.0b013e31823f429f

34. Elangovan S, Pathania R, Ramachandran S, Ananth S, Padia RN, Srinivas SR, Babu E, Hawthorn L, Schoenlein PV, Boettger T, Smith SB, Prasad PD, Ganapathy V, Thangaraju M (2013) Molecular mechanism of SLC5A8 inactivation in breast cancer. Molecular and cellular biology 33 (19):3920-3935. doi:10.1128/mcb.01702-12

35. Park JY, Kim D, Yang M, Park HY, Lee SH, Rincon M, Kreahling J, Plass C, Smiraglia DJ, Tockman MS, Kim SJ (2013) Gene silencing of SLC5A8 identified by genome-wide methylation profiling in lung cancer. Lung cancer (Amsterdam, Netherlands) 79 (3):198-204. doi:10.1016/j.lungcan.2012.11.019

36. Thangaraju M, Gopal E, Martin PM, Ananth S, Smith SB, Prasad PD, Sterneck E, Ganapathy V (2006) SLC5A8 triggers tumor cell apoptosis through pyruvate-dependent inhibition of histone deacetylases. Cancer research 66 (24):11560-11564. doi:10.1158/0008-5472.Can-06-1950

37. Kim JT, Li C, Weiss HL, Zhou Y, Liu C, Wang Q, Evers BM (2019) Regulation of Ketogenic Enzyme HMGCS2 by Wnt/β-catenin/PPARγ Pathway in Intestinal Cells. Cells 8 (9). doi:10.3390/cells8091106

38. Tang D, Zhao YC, Liu H, Luo S, Clarke JM, Glass C, Su L, Shen S, Christiani DC, Gao W, Wei Q (2020) Potentially functional genetic variants in PLIN2, SULT2A1 and UGT1A9 genes of the ketone pathway and survival of nonsmall cell lung cancer. International journal of cancer Journal international du cancer 147 (6):1559-1570. doi:10.1002/ijc.32932

39. Sim J, Johnson RS (2015) Through a Clear Cell, Darkly: HIF2α/PLIN2-Maintained Fat Droplets Protect ccRCCs from ER Stress. Cancer discovery 5 (6):584-585. doi:10.1158/2159-8290.Cd-15-0480

40. Qiu B, Ackerman D, Sanchez DJ, Li B, Ochocki JD, Grazioli A, Bobrovnikova-Marjon E, Diehl JA, Keith B, Simon MC (2015) HIF2α-Dependent Lipid Storage Promotes Endoplasmic Reticulum Homeostasis in Clear-Cell Renal Cell Carcinoma. Cancer discovery 5 (6):652-667. doi:10.1158/2159-8290.Cd-14-1507

41. Cao Q, Ruan H, Wang K, Song Z, Bao L, Xu T, Xiao H, Wang C, Cheng G, Tong J, Meng X, Liu D, Yang H, Chen K, Zhang X (2018) Overexpression of PLIN2 is a prognostic marker and attenuates tumor progression in clear cell renal cell carcinoma. International journal of oncology 53 (1):137-147. doi:10.3892/ijo.2018.4384

42. Okeigwe I, Bulun S, Liu S, Rademaker AW, Coon JS, Kujawa S, Robins J, Yin P (2019) PLIN2 Functions As a Novel Link Between Progesterone Signaling and Metabolism in Uterine Leiomyoma Cells. The Journal of clinical endocrinology and metabolism 104 (12):6256-6264. doi:10.1210/jc.2019-00762

43. Sun C, Luan S, Zhang G, Wang N, Shao H, Luan C (2017) CEBPA-mediated upregulation of the lncRNA PLIN2 promotes the development of chronic myelogenous leukemia via the GSK3 and Wnt/β-catenin signaling pathways. American journal of cancer research 7 (5):1054-1067

44. Okamura Y, Nomoto S, Hayashi M, Hishida M, Nishikawa Y, Yamada S, Fujii T, Sugimoto H, Takeda S, Kodera Y, Nakao A (2011) Identification of the bleomycin hydrolase gene as a methylated tumor suppressor gene in hepatocellular carcinoma using a novel triple-combination array method. Cancer letters 312 (2):150-157. doi:10.1016/j.canlet.2011.07.028

45. de Haas EC, Zwart N, Meijer C, Nuver J, Boezen HM, Suurmeijer AJ, Hoekstra HJ, van der Steege G, Sleijfer DT, Gietema JA (2008) Variation in bleomycin hydrolase gene is associated with reduced survival after chemotherapy for testicular germ cell cancer. Journal of clinical oncology : official journal of the American Society of Clinical Oncology 26 (11):1817-1823. doi:10.1200/jco.2007.14.1606

46. Emmink BL, Verheem A, Van Houdt WJ, Steller EJ, Govaert KM, Pham TV, Piersma SR, Borel Rinkes IH, Jimenez CR, Kranenburg O (2013) The secretome of colon cancer stem cells contains drug-metabolizing enzymes. Journal of proteomics 91:84-96. doi:10.1016/j.jprot.2013.06.027

47. Röhl J, West ZE, Rudolph M, Zaharia A, Van Lonkhuyzen D, Hickey DK, Semmler ABT, Murray RZ (2019) Invasion by activated macrophages requires delivery of nascent membrane-type-1 matrix metalloproteinase through late endosomes/lysosomes to the cell surface. Traffic (Copenhagen, Denmark) 20 (9):661-673. doi:10.1111/tra.12675

48. Althubiti M, Lezina L, Carrera S, Jukes-Jones R, Giblett SM, Antonov A, Barlev N, Saldanha GS, Pritchard CA, Cain K, Macip S (2014) Characterization of novel markers of senescence and their prognostic potential in cancer. Cell death & disease 5 (11):e1528. doi:10.1038/cddis.2014.489

49. Dolai S, Liang T, Orabi AI, Holmyard D, Xie L, Greitzer-Antes D, Kang Y, Xie H, Javed TA, Lam PP, Rubin DC, Thorn P, Gaisano HY (2018) Pancreatitis-Induced Depletion of Syntaxin 2 Promotes Autophagy and Increases Basolateral Exocytosis. Gastroenterology 154 (6):1805-1821.e1805. doi:10.1053/j.gastro.2018.01.025

50. Brasher MI, Martynowicz DM, Grafinger OR, Hucik A, Shanks-Skinner E, Uniacke J, Coppolino MG (2017) Interaction of Munc18c and syntaxin4 facilitates invadopodium formation and extracellular matrix invasion of tumor cells. The Journal of biological chemistry 292 (39):16199-16210. doi:10.1074/jbc.M117.807438

51. Gangwani L, Mikrut M, Galcheva-Gargova Z, Davis RJ (1998) Interaction of ZPR1 with translation elongation factor-1alpha in proliferating cells. J Cell Biol 143 (6):1471-1484. doi:10.1083/jcb.143.6.1471

52. Li X, Chen N, Zhou L, Wang C, Wen X, Jia L, Cui J, Hoffman AR, Hu JF, Li W (2019) Genome-wide target interactome profiling reveals a novel EEF1A1 epigenetic pathway for oncogenic lncRNA MALAT1 in breast cancer. American journal of cancer research 9 (4):714-729

53. Liu S, Hausmann S, Carlson SM, Fuentes ME, Francis JW, Pillai R, Lofgren SM, Hulea L, Tandoc K, Lu J, Li A, Nguyen ND, Caporicci M, Kim MP, Maitra A, Wang H, Wistuba, II, Porco JA, Jr., Bassik MC, Elias JE, Song J, Topisirovic I, Van Rechem C, Mazur PK, Gozani O (2019) METTL13 Methylation of eEF1A Increases Translational Output to Promote Tumorigenesis. Cell 176 (3):491-504.e421. doi:10.1016/j.cell.2018.11.038

54. Liu X, Chen L, Ge J, Yan C, Huang Z, Hu J, Wen C, Li M, Huang D, Qiu Y, Hao H, Yuan R, Lei J, Yu X, Shao J (2016) The Ubiquitin-like Protein FAT10 Stabilizes eEF1A1 Expression to Promote Tumor Proliferation in a Complex Manner. Cancer research 76 (16):4897-4907. doi:10.1158/0008-5472.Can-15-3118

55. Scaggiante B, Dapas B, Bonin S, Grassi M, Zennaro C, Farra R, Cristiano L, Siracusano S, Zanconati F, Giansante C, Grassi G (2012) Dissecting the expression of EEF1A1/2 genes in human prostate cancer cells: the potential of EEF1A2 as a hallmark for prostate transformation and progression. British journal of cancer 106 (1):166-173. doi:10.1038/bjc.2011.500

56. Tomlinson VA, Newbery HJ, Bergmann JH, Boyd J, Scott D, Wray NR, Sellar GC, Gabra H, Graham A, Williams AR, Abbott CM (2007) Expression of eEF1A2 is associated with clear cell histology in ovarian carcinomas: overexpression of the gene is not dependent on modifications at the EEF1A2 locus. British journal of cancer 96 (10):1613-1620. doi:10.1038/sj.bjc.6603748

57. Shi J, Zhang G, Yao D, Liu W, Wang N, Ji M, He N, Shi B, Hou P (2012) Prognostic significance of aberrant gene methylation in gastric cancer. American journal of cancer research 2 (1):116-129

58. Li J, Wang J, Chen Y, Yang L, Chen S (2017) A prognostic 4-gene expression signature for squamous cell lung carcinoma. Journal of cellular physiology 232 (12):3702-3713. doi:10.1002/jcp.25846

59. Golomb L, Bublik DR, Wilder S, Nevo R, Kiss V, Grabusic K, Volarevic S, Oren M (2012) Importin 7 and exportin 1 link c-Myc and p53 to regulation of ribosomal biogenesis. Mol Cell 45 (2):222-232. doi:10.1016/j.molcel.2011.11.022

60. Ju JH, Yang W, Lee KM, Oh S, Nam K, Shim S, Shin SY, Gye MC, Chu IS, Shin I (2013) Regulation of cell proliferation and migration by keratin19-induced nuclear import of early growth response-1 in breast cancer cells. Clinical cancer research : an official journal of the American Association for Cancer Research 19 (16):4335-4346. doi:10.1158/1078-0432.Ccr-12-3295

61. Szczyrba J, Nolte E, Hart M, Döll C, Wach S, Taubert H, Keck B, Kremmer E, Stöhr R, Hartmann A, Wieland W, Wullich B, Grässer FA (2013) Identification of ZNF217, hnRNP-K, VEGF-A and IPO7 as targets for microRNAs that are downregulated in prostate carcinoma. International journal of cancer Journal international du cancer 132 (4):775-784. doi:10.1002/ijc.27731

62. Xue J, Zhou A, Tan C, Wu Y, Lee HT, Li W, Xie K, Huang S (2015) Forkhead Box M1 Is Essential for Nuclear Localization of Glioma-associated Oncogene Homolog 1 in Glioblastoma Multiforme Cells by Promoting Importin-7 Expression. The Journal of biological chemistry 290 (30):18662-18670. doi:10.1074/jbc.M115.662882

63. Roy CN, Andrews NC (2001) Recent advances in disorders of iron metabolism: mutations, mechanisms and modifiers. Human molecular genetics 10 (20):2181-2186. doi:10.1093/hmg/10.20.2181

64. Kory N, Wyant GA, Prakash G, Uit de Bos J, Bottanelli F, Pacold ME, Chan SH, Lewis CA, Wang T, Keys HR, Guo YE, Sabatini DM (2018) SFXN1 is a mitochondrial serine transporter required for one-carbon metabolism. Science 362 (6416). doi:10.1126/science.aat9528

65. Jiang H, Xu S, Chen C (2020) A ten-gene signature-based risk assessment model predicts the prognosis of lung adenocarcinoma. BMC cancer 20 (1):782. doi:10.1186/s12885-020-07235-z

66. Ohara K, Arai E, Takahashi Y, Ito N, Shibuya A, Tsuta K, Kushima R, Tsuda H, Ojima H, Fujimoto H, Watanabe SI, Katai H, Kinoshita T, Shibata T, Kohno T, Kanai Y (2017) Genes involved in development and differentiation are commonly methylated in cancers derived from multiple organs: a single-institutional methylome analysis using 1007 tissue specimens. Carcinogenesis 38 (3):241-251. doi:10.1093/carcin/bgw209

67. Zhu Y, Wang Q, Xia Y, Xiong X, Weng S, Ni H, Ye Y, Chen L, Lin J, Chen Y, Niu H, Chen X, Lin Y (2020) Evaluation of MiR-1908-3p as a novel serum biomarker for breast cancer and analysis its oncogenic function and target genes. BMC cancer 20 (1):644. doi:10.1186/s12885-020-07125-4

68. Sorli SC, Colié S, Albinet V, Dubrac A, Touriol C, Guilbaud N, Bedia C, Fabriàs G, Casas J, Ségui B, Levade T, Andrieu-Abadie N (2013) The nonlysosomal β-glucosidase GBA2 promotes endoplasmic reticulum stress and impairs tumorigenicity of human melanoma cells. FASEB journal : official publication of the Federation of American Societies for Experimental Biology 27 (2):489-498. doi:10.1096/fj.12-215152

69. Astudillo L, Therville N, Colacios C, Ségui B, Andrieu-Abadie N, Levade T (2016) Glucosylceramidases and malignancies in mammals. Biochimie 125:267-280. doi:10.1016/j.biochi.2015.11.009

70. Gaudet P, Livstone MS, Lewis SE, Thomas PD (2011) Phylogenetic-based propagation of functional annotations within the Gene Ontology consortium. Briefings in bioinformatics 12 (5):449-462. doi:10.1093/bib/bbr042

71. Wang VW, Laborde RR, Asmann YW, Li Y, Ma J, Eckloff BW, Tombers NM, Olsen SM, Moore EJ, Olsen KD, Smith DI (2013) Search for chromosome rearrangements: new approaches toward discovery of novel translocations in head and neck squamous cell carcinoma. Head & neck 35 (6):831-835. doi:10.1002/hed.23037

72. Takata R, Takahashi A, Fujita M, Momozawa Y, Saunders EJ, Yamada H, Maejima K, Nakano K, Nishida Y, Hishida A, Matsuo K, Wakai K, Yamaji T, Sawada N, Iwasaki M, Tsugane S, Sasaki M, Shimizu A, Tanno K, Minegishi N, Suzuki K, Matsuda K, Kubo M, Inazawa J, Egawa S, Haiman CA, Ogawa O, Obara W, Kamatani Y, Akamatsu S, Nakagawa H (2019) 12 new susceptibility loci for prostate cancer identified by genome-wide association study in Japanese population. Nature communications 10 (1):4422. doi:10.1038/s41467-019-12267-6

73. Zhang X, Zhang Y, Miao Y, Zhou H, Jiang G, Wang E (2017) TMEM17 depresses invasion and metastasis in lung cancer cells via ERK signaling pathway. Oncotarget 8 (41):70685-70694. doi:10.18632/oncotarget.19977

74. Zhao Y, Song K, Zhang Y, Xu H, Zhang X, Wang L, Fan C, Jiang G, Wang E (2018) TMEM17 promotes malignant progression of breast cancer via AKT/GSK3β signaling. Cancer management and research 10:2419-2428. doi:10.2147/cmar.S168723

75. Choi M, Kadara H, Zhang J, Parra ER, Rodriguez-Canales J, Gaffney SG, Zhao Z, Behrens C, Fujimoto J, Chow C, Kim K, Kalhor N, Moran C, Rimm D, Swisher S, Gibbons DL, Heymach J, Kaftan E, Townsend JP, Lynch TJ, Schlessinger J, Lee J, Lifton RP, Herbst RS, Wistuba, II (2017) Mutation profiles in early-stage lung squamous cell carcinoma with clinical follow-up and correlation with markers of immune function. Annals of oncology : official journal of the European Society for Medical Oncology / ESMO 28 (1):83-89. doi:10.1093/annonc/mdw437

76. Shen-Gunther J, Wang CM, Poage GM, Lin CL, Perez L, Banks NA, Huang TH (2016) Molecular Pap smear: HPV genotype and DNA methylation of ADCY8, CDH8, and ZNF582 as an integrated biomarker for high-grade cervical cytology. Clinical epigenetics 8 (1):96. doi:10.1186/s13148-016-0263-9

77. Warrington NM, Sun T, Luo J, McKinstry RC, Parkin PC, Ganzhorn S, Spoljaric D, Albers AC, Merkelson A, Stewart DR, Stevenson DA, Viskochil D, Druley TE, Forys JT, Reilly KM, Fisher MJ, Tabori U, Allen JC, Schiffman JD, Gutmann DH, Rubin JB (2015) The cyclic AMP pathway is a sex-specific modifier of glioma risk in type I neurofibromatosis patients. Cancer research 75 (1):16-21. doi:10.1158/0008-5472.Can-14-1891

78. Orchel J, Witek L, Kimsa M, Strzalka-Mrozik B, Kimsa M, Olejek A, Mazurek U (2012) Expression patterns of kinin-dependent genes in endometrial cancer. International journal of gynecological cancer : official journal of the International Gynecological Cancer Society 22 (6):937-944. doi:10.1097/IGC.0b013e318259d8da

79. Bonne A, Vreede L, Kuiper RP, Bodmer D, Jansen C, Eleveld M, van Erp F, Arkesteijn G, Hoogerbrugge N, van Ravenswaaij C, Schoenmakers EF, Geurts van Kessel A (2007) Mapping of constitutional translocation breakpoints in renal cell cancer patients: identification of KCNIP4 as a candidate gene. Cancer genetics and cytogenetics 179 (1):11-18. doi:10.1016/j.cancergencyto.2007.07.005

80. Brenner DR, Amos CI, Brhane Y, Timofeeva MN, Caporaso N, Wang Y, Christiani DC, Bickeböller H, Yang P, Albanes D, Stevens VL, Gapstur S, McKay J, Boffetta P, Zaridze D, Szeszenia-Dabrowska N, Lissowska J, Rudnai P, Fabianova E, Mates D, Bencko V, Foretova L, Janout V, Krokan HE, Skorpen F, Gabrielsen ME, Vatten L, Njølstad I, Chen C, Goodman G, Lathrop M, Vooder T, Välk K, Nelis M, Metspalu A, Broderick P, Eisen T, Wu X, Zhang D, Chen W, Spitz MR, Wei Y, Su L, Xie D, She J, Matsuo K, Matsuda F, Ito H, Risch A, Heinrich J, Rosenberger A, Muley T, Dienemann H, Field JK, Raji O, Chen Y, Gosney J, Liloglou T, Davies MP, Marcus M, McLaughlin J, Orlow I, Han Y, Li Y, Zong X, Johansson M, Liu G, Tworoger SS, Le Marchand L, Henderson BE, Wilkens LR, Dai J, Shen H, Houlston RS, Landi MT, Brennan P, Hung RJ (2015) Identification of lung cancer histology-specific variants applying Bayesian framework variant prioritization approaches within the TRICL and ILCCO consortia. Carcinogenesis 36 (11):1314-1326. doi:10.1093/carcin/bgv128

81. Poirier JG, Brennan P, McKay JD, Spitz MR, Bickeböller H, Risch A, Liu G, Le Marchand L, Tworoger S, McLaughlin J, Rosenberger A, Heinrich J, Brüske I, Muley T, Henderson BE, Wilkens LR, Zong X, Li Y, Hao K, Timens W, Bossé Y, Sin DD, Obeidat M, Amos CI, Hung RJ (2015) Informed genome-wide association analysis with family history as a secondary phenotype identifies novel loci of lung cancer. Genetic epidemiology 39 (3):197-206. doi:10.1002/gepi.21882

82. Zhu S, Ye L, Bennett S, Xu H, He D, Xu J (2020) Molecular structure and function of microfibrillar-associated proteins in skeletal and metabolic disorders and cancers. Journal of cellular physiology. doi:10.1002/jcp.29893

83. Cao Q, Gery S, Dashti A, Yin D, Zhou Y, Gu J, Koeffler HP (2009) A role for the clock gene per1 in prostate cancer. Cancer research 69 (19):7619-7625. doi:10.1158/0008-5472.Can-08-4199

84. Guo X, Li K, Jiang W, Hu Y, Xiao W, Huang Y, Feng Y, Pan Q, Wan R (2020) RNA demethylase ALKBH5 prevents pancreatic cancer progression by posttranscriptional activation of PER1 in an m6A-YTHDF2-dependent manner. Molecular cancer 19 (1):91. doi:10.1186/s12943-020-01158-w

85. Han Y, Meng F, Venter J, Wu N, Wan Y, Standeford H, Francis H, Meininger C, Greene J, Jr., Trzeciakowski JP, Ehrlich L, Glaser S, Alpini G (2016) miR-34a-dependent overexpression of Per1 decreases cholangiocarcinoma growth. Journal of hepatology 64 (6):1295-1304. doi:10.1016/j.jhep.2016.02.024

86. Hernández-Rosas F, Hernández-Oliveras A, Flores-Peredo L, Rodríguez G, Zarain-Herzberg Á, Caba M, Santiago-García J (2018) Histone deacetylase inhibitors induce the expression of tumor suppressor genes Per1 and Per2 in human gastric cancer cells. Oncology letters 16 (2):1981-1990. doi:10.3892/ol.2018.8851

87. Cepeda D, Ng HF, Sharifi HR, Mahmoudi S, Cerrato VS, Fredlund E, Magnusson K, Nilsson H, Malyukova A, Rantala J, Klevebring D, Viñals F, Bhaskaran N, Zakaria SM, Rahmanto AS, Grotegut S, Nielsen ML, Szigyarto CA, Sun D, Lerner M, Navani S, Widschwendter M, Uhlén M, Jirström K, Pontén F, Wohlschlegel J, Grandér D, Spruck C, Larsson LG, Sangfelt O (2013) CDK-mediated activation of the SCF(FBXO) (28) ubiquitin ligase promotes MYC-driven transcription and tumourigenesis and predicts poor survival in breast cancer. EMBO molecular medicine 5 (7):1067-1086. doi:10.1002/emmm.201202341

88. De Melo J, Kim SS, Lourenco C, Penn LZ (2017) Lysine-52 stabilizes the MYC oncoprotein through an SCF(Fbxw7)-independent mechanism. Oncogene 36 (49):6815-6822. doi:10.1038/onc.2017.268

89. Park C, Lee Y, Je S, Chang S, Kim N, Jeong E, Yoon S (2019) Overexpression and Selective Anticancer Efficacy of ENO3 in STK11 Mutant Lung Cancers. Molecules and cells 42 (11):804-809. doi:10.14348/molcells.2019.0099

90. Chakraborty S, Jiang C, Gau D, Oddo M, Ding Z, Vollmer L, Joy M, Schiemann W, Stolz DB, Vogt A, Ghosh S, Roy P (2018) Profilin-1 deficiency leads to SMAD3 upregulation and impaired 3D outgrowth of breast cancer cells. British journal of cancer 119 (9):1106-1117. doi:10.1038/s41416-018-0284-6

91. Coumans JV, Gau D, Poljak A, Wasinger V, Roy P, Moens PD (2014) Profilin-1 overexpression in MDA-MB-231 breast cancer cells is associated with alterations in proteomics biomarkers of cell proliferation, survival, and motility as revealed by global proteomics analyses. Omics : a journal of integrative biology 18 (12):778-791. doi:10.1089/omi.2014.0075

92. Yao W, Ji S, Qin Y, Yang J, Xu J, Zhang B, Xu W, Liu J, Shi S, Liu L, Liu C, Long J, Ni Q, Li M, Yu X (2014) Profilin-1 suppresses tumorigenicity in pancreatic cancer through regulation of the SIRT3-HIF1α axis. Molecular cancer 13:187. doi:10.1186/1476-4598-13-187

93. Ding Z, Joy M, Bhargava R, Gunsaulus M, Lakshman N, Miron-Mendoza M, Petroll M, Condeelis J, Wells A, Roy P (2014) Profilin-1 downregulation has contrasting effects on early vs late steps of breast cancer metastasis. Oncogene 33 (16):2065-2074. doi:10.1038/onc.2013.166

94. Schoppmeyer R, Zhao R, Cheng H, Hamed M, Liu C, Zhou X, Schwarz EC, Zhou Y, Knörck A, Schwär G, Ji S, Liu L, Long J, Helms V, Hoth M, Yu X, Qu B (2017) Human profilin 1 is a negative regulator of CTL mediated cell-killing and migration. European journal of immunology 47 (9):1562-1572. doi:10.1002/eji.201747124

95. Toma VA, Tigu AB, Farcaș AD, Sevastre B, Taulescu M, Gherman AMR, Roman I, Fischer-Fodor E, Pârvu M (2019) New Aspects Towards a Molecular Understanding of the Allicin Immunostimulatory Mechanism via Colec12, MARCO, and SCARB1 Receptors. International journal of molecular sciences 20 (15). doi:10.3390/ijms20153627

96. Zhao C, Putnik M, Gustafsson JA, Dahlman-Wright K (2009) Microarray analysis of altered gene expression in ERbeta-overexpressing HEK293 cells. Endocrine 36 (2):224-232. doi:10.1007/s12020-009-9233-8

97. Tan Y, Hu X, Deng Y, Yuan P, Xie Y, Wang J (2018) TRA2A promotes proliferation, migration, invasion and epithelial mesenchymal transition of glioma cells. Brain research bulletin 143:138-144. doi:10.1016/j.brainresbull.2018.10.006

98. Liu T, Sun H, Zhu D, Dong X, Liu F, Liang X, Chen C, Shao B, Wang M, Wang Y, Sun B (2017) TRA2A Promoted Paclitaxel Resistance and Tumor Progression in Triple-Negative Breast Cancers via Regulating Alternative Splicing. Molecular cancer therapeutics 16 (7):1377-1388. doi:10.1158/1535-7163.Mct-17-0026

99. Nguyen TM, Kabotyanski EB, Reineke LC, Shao J, Xiong F, Lee JH, Dubrulle J, Johnson H, Stossi F, Tsoi PS, Choi KJ, Ellis AG, Zhao N, Cao J, Adewunmi O, Ferreon JC, Ferreon ACM, Neilson JR, Mancini MA, Chen X, Kim J, Ma L, Li W, Rosen JM (2020) The SINEB1 element in the long non-coding RNA Malat1 is necessary for TDP-43 proteostasis. Nucleic acids research 48 (5):2621-2642. doi:10.1093/nar/gkz1176

100. El Ouardi D, Idrissou M, Sanchez A, Penault-Llorca F, Bignon YJ, Guy L, Bernard-Gallon D (2020) The Inhibition of the Histone Methyltransferase EZH2 by DZNEP or SiRNA Demonstrates Its Involvement in MGMT, TRA2A, RPS6KA2, and U2AF1 Gene Regulation in Prostate Cancer. Omics : a journal of integrative biology 24 (2):116-118. doi:10.1089/omi.2019.0162

101. Ngollo M, Lebert A, Daures M, Judes G, Rifai K, Dubois L, Kemeny JL, Penault-Llorca F, Bignon YJ, Guy L, Bernard-Gallon D (2017) Global analysis of H3K27me3 as an epigenetic marker in prostate cancer progression. BMC cancer 17 (1):261. doi:10.1186/s12885-017-3256-y

102. An S, Huang W, Huang X, Cun Y, Cheng W, Sun X, Ren Z, Chen Y, Chen W, Wang J (2020) Integrative network analysis identifies cell-specific trans regulators of m6A. Nucleic acids research 48 (4):1715-1729. doi:10.1093/nar/gkz1206

103. Duan C, Wang H, Chen Y, Chu P, Xing T, Gao C, Yue Z, Zheng J, Jin M, Gu W, Ma X (2018) Whole exome sequencing reveals novel somatic alterations in neuroblastoma patients with chemotherapy. Cancer cell international 18:21. doi:10.1186/s12935-018-0521-3

104. Maekawa M, Higashiyama S (2020) KCTD10 Biology: An Adaptor for the Ubiquitin E3 Complex Meets Multiple Substrates: Emerging Divergent Roles of the cullin-3/KCTD10 E3 Ubiquitin Ligase Complex in Various Cell Lines. BioEssays : news and reviews in molecular, cellular and developmental biology 42 (8):e1900256. doi:10.1002/bies.201900256

105. Maekawa M, Hiyoshi H, Nakayama J, Kido K, Sawasaki T, Semba K, Kubota E, Joh T, Higashiyama S (2019) Cullin-3/KCTD10 complex is essential for K27-polyubiquitination of EIF3D in human hepatocellular carcinoma HepG2 cells. Biochemical and biophysical research communications 516 (4):1116-1122. doi:10.1016/j.bbrc.2019.07.010

106. Murakami A, Maekawa M, Kawai K, Nakayama J, Araki N, Semba K, Taguchi T, Kamei Y, Takada Y, Higashiyama S (2019) Cullin-3/KCTD10 E3 complex is essential for Rac1 activation through RhoB degradation in human epidermal growth factor receptor 2-positive breast cancer cells. Cancer science 110 (2):650-661. doi:10.1111/cas.13899

107. Wang Y, Zheng Y, Luo F, Fan X, Chen J, Zhang C, Hui R (2009) KCTD10 interacts with proliferating cell nuclear antigen and its down-regulation could inhibit cell proliferation. Journal of cellular biochemistry 106 (3):409-413. doi:10.1002/jcb.22026

108. Gervin E, Shin B, Opperman R, Cullen M, Feser R, Maiti S, Majumder M (2020) Chemically Induced Hypoxia Enhances miRNA Functions in Breast Cancer. Cancers 12 (8). doi:10.3390/cancers12082008

109. Liu C, Banister CE, Buckhaults PJ (2019) Spindle Assembly Checkpoint Inhibition Can Resensitize p53-Null Stem Cells to Cancer Chemotherapy. Cancer research 79 (9):2392-2403. doi:10.1158/0008-5472.Can-18-3024

110. Yu DH, Tang L, Dong H, Dong Z, Zhang L, Fu J, Su X, Zhang T, Fu H, Han L, Xie L, Chen H, Qian Z, Zhu G, Wang J, Ye Q, Zhang J, Yin X, Zhang X, Ji J, Ji Q (2015) Oncogenic HER2 fusions in gastric cancer. Journal of translational medicine 13:116. doi:10.1186/s12967-015-0476-2

111. Gorroño-Etxebarria I, Aguirre U, Sanchez S, González N, Escobar A, Zabalza I, Quintana JM, Vivanco MD, Waxman J, Kypta RM (2019) Wnt-11 as a Potential Prognostic Biomarker and Therapeutic Target in Colorectal Cancer. Cancers 11 (7). doi:10.3390/cancers11070908

112. Murillo-Garzón V, Gorroño-Etxebarria I, Åkerfelt M, Puustinen MC, Sistonen L, Nees M, Carton J, Waxman J, Kypta RM (2018) Frizzled-8 integrates Wnt-11 and transforming growth factor-β signaling in prostate cancer. Nature communications 9 (1):1747. doi:10.1038/s41467-018-04042-w

113. Hoffmann S, Pentakota S, Mund A, Haahr P, Coscia F, Gallo M, Mann M, Taylor NM, Mailand N (2020) FAM111 protease activity undermines cellular fitness and is amplified by gain-of-function mutations in human disease. EMBO reports:e50662. doi:10.15252/embr.202050662

114. Kawasaki K, Nojima S, Hijiki S, Tahara S, Ohshima K, Matsui T, Hori Y, Kurashige M, Umeda D, Kiyokawa H, Kido K, Okuzaki D, Morii E (2020) FAM111B enhances proliferation of KRAS-driven lung adenocarcinoma by degrading p16. Cancer science 111 (7):2635-2646. doi:10.1111/cas.14483

115. Dutta S, Roy S, Polavaram NS, Stanton MJ, Zhang H, Bhola T, Hönscheid P, Donohue TM, Jr., Band H, Batra SK, Muders MH, Datta K (2016) Neuropilin-2 Regulates Endosome Maturation and EGFR Trafficking to Support Cancer Cell Pathobiology. Cancer research 76 (2):418-428. doi:10.1158/0008-5472.Can-15-1488

116. Mercurio AM (2019) VEGF/Neuropilin Signaling in Cancer Stem Cells. International journal of molecular sciences 20 (3). doi:10.3390/ijms20030490

117. Elaimy AL, Amante JJ, Zhu LJ, Wang M, Walmsley CS, FitzGerald TJ, Goel HL, Mercurio AM (2019) The VEGF receptor neuropilin 2 promotes homologous recombination by stimulating YAP/TAZ-mediated Rad51 expression. Proceedings of the National Academy of Sciences of the United States of America 116 (28):14174-14180. doi:10.1073/pnas.1821194116

118. Borkowetz A, Froehner M, Rauner M, Conrad S, Erdmann K, Mayr T, Datta K, Hofbauer LC, Baretton GB, Wirth M, Fuessel S, Toma M, Muders MH (2020) Neuropilin-2 is an independent prognostic factor for shorter cancer-specific survival in patients with acinar adenocarcinoma of the prostate. International journal of cancer Journal international du cancer 146 (9):2619-2627. doi:10.1002/ijc.32679

119. Roy S, Bag AK, Dutta S, Polavaram NS, Islam R, Schellenburg S, Banwait J, Guda C, Ran S, Hollingsworth MA, Singh RK, Talmadge JE, Muders MH, Batra SK, Datta K (2018) Macrophage-Derived Neuropilin-2 Exhibits Novel Tumor-Promoting Functions. Cancer research 78 (19):5600-5617. doi:10.1158/0008-5472.Can-18-0562

120. Kennedy E, Everson TM, Punshon T, Jackson BP, Hao K, Lambertini L, Chen J, Karagas MR, Marsit CJ (2020) Copper associates with differential methylation in placentae from two US birth cohorts. Epigenetics 15 (3):215-230. doi:10.1080/15592294.2019.1661211

121. Wang L, Yang J, Huang J, Wen ZQ, Xu N, Liu X, Zhang JH, Li WL (2020) miRNA Expression Profile in the N2 Phenotype Neutrophils of Colorectal Cancer and Screen of Putative Key miRNAs. Cancer management and research 12:5491-5503. doi:10.2147/cmar.S251427

122. Shiwaku H, Yoshimura N, Tamura T, Sone M, Ogishima S, Watase K, Tagawa K, Okazawa H (2010) Suppression of the novel ER protein Maxer by mutant ataxin-1 in Bergman glia contributes to non-cell-autonomous toxicity. The EMBO journal 29 (14):2446-2460. doi:10.1038/emboj.2010.116

123. Kwon J, Cho HJ, Han SH, No JG, Kwon JY, Kim H (2010) A novel LZAP-binding protein, NLBP, inhibits cell invasion. The Journal of biological chemistry 285 (16):12232-12240. doi:10.1074/jbc.M109.065920

124. Li C, Han T, Guo R, Chen P, Peng C, Prag G, Hu R (2020) An Integrative Synthetic Biology Approach to Interrogating Cellular Ubiquitin and Ufm Signaling. International journal of molecular sciences 21 (12). doi:10.3390/ijms21124231

125. Liu J, Guan D, Dong M, Yang J, Wei H, Liang Q, Song L, Xu L, Bai J, Liu C, Mao J, Zhang Q, Zhou J, Wu X, Wang M, Cong YS (2020) UFMylation maintains tumour suppressor p53 stability by antagonizing its ubiquitination. Nature cell biology. doi:10.1038/s41556-020-0559-z

126. Qin B, Yu J, Nowsheen S, Wang M, Tu X, Liu T, Li H, Wang L, Lou Z (2019) UFL1 promotes histone H4 ufmylation and ATM activation. Nature communications 10 (1):1242. doi:10.1038/s41467-019-09175-0

127. Zhang M, Zhu X, Zhang Y, Cai Y, Chen J, Sivaprakasam S, Gurav A, Pi W, Makala L, Wu J, Pace B, Tuan-Lo D, Ganapathy V, Singh N, Li H (2015) RCAD/Ufl1, a Ufm1 E3 ligase, is essential for hematopoietic stem cell function and murine hematopoiesis. Cell death and differentiation 22 (12):1922-1934. doi:10.1038/cdd.2015.51

128. Zhang Y, Yu C (2020) Prognostic characterization of OAS1/OAS2/OAS3/OASL in breast cancer. BMC cancer 20 (1):575. doi:10.1186/s12885-020-07034-6

129. Zhang L, Jiang Y, Lu X, Zhao H, Chen C, Wang Y, Hu W, Zhu Y, Yan H, Yan F (2019) Genomic characterization of cervical cancer based on human papillomavirus status. Gynecologic oncology 152 (3):629-637. doi:10.1016/j.ygyno.2018.12.017

130. Néant I, Haiech J, Kilhoffer MC, Aulestia FJ, Moreau M, Leclerc C (2018) Ca(2+)-Dependent Transcriptional Repressors KCNIP and Regulation of Prognosis Genes in Glioblastoma. Frontiers in molecular neuroscience 11:472. doi:10.3389/fnmol.2018.00472

131. Orlando G, Law PJ, Palin K, Tuupanen S, Gylfe A, Hänninen UA, Cajuso T, Tanskanen T, Kondelin J, Kaasinen E, Sarin AP, Kaprio J, Eriksson JG, Rissanen H, Knekt P, Pukkala E, Jousilahti P, Salomaa V, Ripatti S, Palotie A, Järvinen H, Renkonen-Sinisalo L, Lepistö A, Böhm J, Mecklin JP, Al-Tassan NA, Palles C, Martin L, Barclay E, Tenesa A, Farrington S, Timofeeva MN, Meyer BF, Wakil SM, Campbell H, Smith CG, Idziaszczyk S, Maughan TS, Kaplan R, Kerr R, Kerr D, Buchanan DD, Win AK, Hopper J, Jenkins M, Lindor NM, Newcomb PA, Gallinger S, Conti D, Schumacher F, Casey G, Taipale J, Cheadle JP, Dunlop MG, Tomlinson IP, Aaltonen LA, Houlston RS (2016) Variation at 2q35 (PNKD and TMBIM1) influences colorectal cancer risk and identifies a pleiotropic effect with inflammatory bowel disease. Human molecular genetics 25 (11):2349-2359. doi:10.1093/hmg/ddw087

132. Wang P, Liu H, Wang Y, Liu O, Zhang J, Gleason A, Yang Z, Wang H, Shi A, Grant BD (2016) RAB-10 Promotes EHBP-1 Bridging of Filamentous Actin and Tubular Recycling Endosomes. PLoS genetics 12 (6):e1006093. doi:10.1371/journal.pgen.1006093
